# Peroxiredoxin 2 regulates DAF-16/FOXO mediated mitochondrial remodelling in response to exercise that is disrupted in ageing

**DOI:** 10.1016/j.molmet.2024.102003

**Published:** 2024-08-06

**Authors:** Qin Xia, Penglin Li, José C. Casas-Martinez, Antonio Miranda-Vizuete, Emma McDermott, Peter Dockery, Katarzyna Goljanek-Whysall, Brian McDonagh

**Affiliations:** 1 Discipline of Physiology, School of Medicine, Ireland; 2 Apoptosis Research Centre, University of Galway, Ireland; 3 Instituto de Biomedicina de Sevilla, Hospital Universitario Virgen del Rocío/CSIC/Universidad de Sevilla, Sevilla, Spain; 4 Centre for Microscopy and Imaging, Discipline of Anatomy, School of Medicine, University of Galway, Ireland; 5 Institute of Lifecourse and Medical Sciences, University of Liverpool, UK

**Keywords:** Ageing, Peroxiredoxin 2, Exercise, *C.elegans*, Mitochondrial ER contact sites, DAF-16, *eat-3*

## Abstract

**Objectives:**

A decline in mitochondrial function and increased susceptibility to oxidative stress is a hallmark of ageing. Exercise endogenously generates reactive oxygen species (ROS) in skeletal muscle and promotes mitochondrial remodelling resulting in improved mitochondrial function. It is unclear how exercise induced redox signalling results in alterations in mitochondrial dynamics and morphology.

**Methods:**

In this study, a *Caenorhabditis elegans* model of exercise and ageing was used to determine the mechanistic role of Peroxiredoxin 2 (PRDX-2) in regulating mitochondrial morphology. Mitochondrial morphology was analysed using transgenic reporter strains and transmission electron microscopy, complimented with the analysis of the effects of ageing and exercise on physiological activity.

**Results:**

The redox state of PRDX-2 was altered with exercise and ageing, hyperoxidised peroxiredoxins were detected in old worms along with basally elevated intracellular ROS. Exercise generated intracellular ROS and rapid mitochondrial remodelling, which was disrupted with age. The exercise intervention promoted mitochondrial ER contact sites (MERCS) assembly and increased DAF-16/FOXO nuclear localisation. The *prdx-2* mutant strain had a disrupted mitochondrial network as evidenced by increased mitochondrial fragmentation. In the *prdx-2* mutant strain, exercise did not activate DAF-16/FOXO, mitophagy or increase MERCS assembly. The results demonstrate that exercise generated ROS increased DAF-16/FOXO transcription factor nuclear localisation required for activation of mitochondrial fusion events that were blunted with age.

**Conclusions:**

The data demonstrate the critical role of PRDX-2 in orchestrating mitochondrial remodelling in response to a physiological stress by regulating redox dependent DAF-16/FOXO nuclear localisation.

## Highlights

• Exercise generates ROS and promotes mitochondrial remodelling dependent on DAF-16.
• Exercise induces mitochondrial ER contact site assembly and mitochondrial dynamics.
• Ageing and loss of PRDX-2 results in disrupted mitochondrial fusion.
• The redox state of PRDX-2 determines appropriate DAF-16 nuclear localisation.

## Graphical abstract

**Figure:**
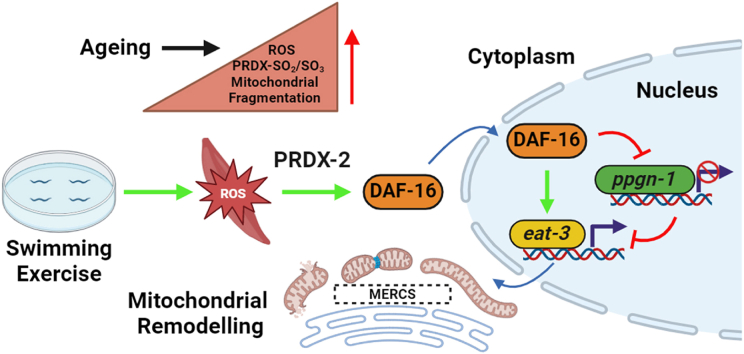

## 1 Introduction

Ageing results in a gradual decline in physiological function, leading to compromised overall health and increased susceptibility to diseases and mortality [1]. Age-related disorders, such as diabetes, sarcopenia and neurodegenerative conditions, pose a significant and growing public health challenge [2,3]. Exercise is recognised as a nonpharmacological intervention to mitigate the adverse effects of ageing. Physical exercise is essential for preserving and enhancing skeletal muscle function, ameliorating age-related muscle atrophy and providing systemic benefits against various age-related diseases [4,5]. During exercise, skeletal muscle mitochondria undergo significant structural remodelling to meet energy and nutritional requirements, impacting cellular metabolism and signalling [4,6]. The decline in mitochondrial function is a hallmark of ageing [7] and the accumulation of dysfunctional mitochondria with age has been observed in *Caenorhabditis elegans* [8]. Damaged or dysfunctional components of mitochondria can be selectively removed via fission and transfer to the lysosome for degradation or mitophagy [9]. Another critical mechanism for mitochondrial health is fusion, where individual mitochondria merge to form a singular, enlarged structure [10]. The maintenance of mitochondrial quality, encompassing processes such as mitochondrial biogenesis, fission and fusion, is critically important in governing both the structure and functionality of mitochondria. In *C. elegans*, mitochondrial fission involves DRP-1 (DRP1 in mammals), while fusion requires FZO-1 (MFN1/2 in mammals) for outer membrane fusion and EAT-3 (OPA1 in mammals) for inner membrane fusion [11]. Overexpression of DRP-1 leads to mitochondrial hyper-fragmentation, whereas suppression of DRP-1 results in fragmented mitochondrial matrices but fused outer mitochondrial membranes [12]. Mitochondrial fusion mutant strains (*eat-3* and *fzo-1*) also display increased mitochondrial fragmentation [13]. Interestingly, overexpression of either mitochondrial fusion (*eat-3* and *fzo-1*) and fission (*drp-1*) genes result in an extension of lifespan despite generating a fragmented mitochondrial network [14].

The Forkhead Box O (FOXO) family of transcription factors play crucial roles in regulating diverse cellular processes such as oxidative stress response, mitochondrial function and cellular metabolism [15]. In *C. elegans*, the FOXO ortholog, DAF-16, exhibits heightened nuclear localisation in response to elevated reactive oxygen species (ROS) and following a swimming exercise protocol [[16], [17], [18]]. Increased nuclear accumulation of DAF-16 following a swimming intervention resulted in autophagy activation and an extension of lifespan but this was not apparent after *daf-16* RNAi [19]. Moreover, DAF-16 has been reported to regulate mitochondrial morphology [18] and restoration of the expression of *daf-16* in *daf-2 daf-16* mutants promotes an increase in mitochondrial mass and function [20]. DAF-16 activity has been reported to alter with age and that DAF-16 age dependent targets of gene expression are different from canonical DAF-16 targets downstream of insulin signalling [21]. The mitochondrial proteases SPG-7 and PPGN-1 target the mitochondrial fusion protein EAT-3, DAF-16 nuclear localisation results in transcriptional repression of *spg-7* and *ppgn-1*, allowing the negative regulation of EAT-3 to be alleviated and thereby promoting mitochondrial fusion [18]. At L4 stage, the mitochondrial morphology of the *daf-16 (mu86)* mutant was reported as similar to wild type but following exercise induced mitochondrial fragmentation, there was a failure to generate elongated mitochondria supporting disrupted mitochondrial fusion [18].

Mitochondrial morphology is also thought to determine fuel substrate use, whereby an increase in mitochondrial fragmentation increases fatty acid oxidation [22]. It was demonstrated that fragmented mitochondria promote Carnitine O-palmitoyltransferase 1 (CPT1) regulated long chain fatty acid oxidation [22]. Our previous proteomic analysis following a swim exercise in wild type (N2) *C. elegans* demonstrated an increased abundance of proteins involved in fatty acids oxidation and decreased abundance of fatty acid anabolism, the most upregulated protein reported was CPT-1 [23]. These results indicated that under conditions of bioenergetic stress such as during exercise, there was a promotion of mitochondrial fatty acid oxidation and increased mitochondrial fragmentation.

Mitochondria and the endoplasmic reticulum (ER) are key regulatory hubs in maintaining cellular homeostasis and have a synergistic relationship that determines their function and adaptability to the cellular environment. Communication between these organelles is mediated by Mitochondrial ER contact sites (MERCS), allowing the exchange of metabolites, lipids and calcium [24,25]. MERCS are dynamic and relatively stable structures between mitochondria and ER (<50 nm) that remodel in response to cellular signalling events, that can affect the function of both organelles. Recent evidence would suggest that during acute ER stress, there is activation of the adaptive Unfolded Protein Response (UPR), resulting in ER and MERCS remodelling [26,27]. MERCS have been identified as regulating the sites of mitochondrial fission and there is some evidence to suggest they also play a role in mitochondrial fusion [25,28]. During fission, the ER constricts mitochondria at the site of fission using Drp1 [29]. Furthermore, the sites of mitochondrial fission have distinct ROS signatures that may regulate formation of MERCS, fission at the periphery or tip results in mitochondrial fragments destined for degradation while midzone fission is preferential for mitochondrial dynamics [30]. Disruption of mitochondrial dynamics has been reported in a wide variety of age-related diseases including neurodegeneration and sarcopenia, associated with an accumulation of dysfunctional mitochondria [31]. An increase in MERCS formation has been proposed to be involved in cell senescence and in models of neurodegenerative disease [32,33]. In skeletal muscle, sarcomeres are surrounded by mitochondria and sarco/endoplasmic reticulum, essential for Ca^2+^ regulation. However, decreased MERCS formation has been reported with age [34]. Energy stress and subsequent AMPK activation has been demonstrated in cell models to promote autophagy and MERCS formation [35]. Introducing an exercise protocol that promotes a mild ER stress response and induces mitochondrial remodelling, will ultimately result in an improved bioenergetic profile. During exercise, there is a site-specific generation of endogenous ROS in skeletal muscle [36,37]. However, it has been reported in a number of different studies that there is a chronic basal elevation of ROS in muscle with age [38,39]. The acute generation of ROS following a physiological stress such as exercise activates specific signalling pathways including NRF2 and NF-κB [40,41], promoting a beneficial adaptive response. However, the majority of prior investigations mainly focused on static redox states, with limited exploration of dynamic responses in both young and elderly individuals to redox stress. The Peroxiredoxins (PRDXs) family, constitute up to 1% of cellular protein content and are generally considered as a group of antioxidant enzymes with peroxidase activity [42]. Peroxiredoxin 2 (PRDX-2) functions as a peroxidase to mitigate ROS during stress and has been demonstrated to be required for the beneficial adaptation to exercise in *C. elegans* [23]. Understanding the specific role of PRDX-2 in regulating mitochondrial dynamics during exercise and ageing is essential.

In this study, the nematode *C. elegans* was utilised as a model system to investigate the intricate interplay between ageing and exercise, with a specific focus on elucidating the role of PRDX-2 in mitochondrial dynamics. We demonstrated that exercise promoted mitochondrial dynamics, MERCS formation and fatigue in *C. elegans*. A 24-h recovery period was adequate to increase mitochondrial fusion in young worms. Old worms exhibited fragmented mitochondria, no or delayed recovery response, an accumulation of basal ROS, an increase in hyperoxidised Peroxiredoxins and decreased fitness. The *prdx-2* mutant strain had increased mitochondrial fragmentation but did not activate mitophagy and displayed reduced physical fitness. Additionally, the acute swim exercise induced nuclear localisation of DAF-16 in the adult wild types promoting mitochondrial fusion. However, this response was not observed with age in the wild type strain or at any age in the *prdx-2* mutant strain, which did not stimulate mitochondrial fusion as a result of a failure to stimulate DAF-16 dependent signalling. Our results demonstrate blunted mitochondrial remodelling and an accumulation of ROS with age associated with disruption in the redox state of PRDX-2, highlighting the crucial role of PRDX-2 in coordinating mitochondrial adaptation in response to exercise and ageing.

## 2 Materials and methods

### 2.1 *C. elegans* strains

*C. elegans* were cultured on NGM plates seeded with E. Coli (OP50) at 20 °C. *C. elegans* stains N2 wild type, CL2166 (*dvIs19[(pAF15)gst-4p::gfp] III)* and SJ4103 (*zcIs14 [myo-3::gfp(mit)]*) strains were obtained from the Caenorhabditis Genetics Center (CGC); IRE2539 (*Ex[pmyo-3 tomm-20::Rosella;unc-119(+)]*) was a gift from the Tavernarakis lab University of Crete, Greece; TJ356 (*zIs356 [daf-16p::daf-16a/b::GFP + rol-6(su1006)*) and OH16024 (*daf-16(ot971[daf-16::GFP]) I*) were obtained from the Miranda-Vizuete lab at the Instituto de Biomedicina de Sevilla. The VE1 (*prdx-2(gk169) II*) strain was obtained from Elizabeth Veal, Newcastle University. In this paper, the *prdx-2* mutant strain was crossed with the reporter strains: SKN-1 reporter CL2166, DAF-16 reporter OH16024/TJ356, mitochondrial GFP reporter SJ4103 and mitophagy reporter IR2539.

### 2.2 *C. elegans* swimming exercise

Adult D1 worms were washed off plates and the larvae were removed daily to obtain worms at different ages (adult D4, D8, D12). For the acute exercise and control groups, 30 worms at least were transferred to unseeded NGM plates with or without M9 buffer, worms were allowed to swim or crawl for a duration of 90 min at D4, D8 and D12 separately [43]. The recovery group underwent a 24-h recovery period following the exercise regimen. For the chronic swimming exercise, adult D1 worms underwent 90-min swimming sessions twice daily over a period of 5 days.

### 2.3 Oxidative stress survival assays

For the paraquat and sodium arsenite stress assays, 10 worms were selected for each group with a total of 60 worms per condition. These worms were carefully placed in individual wells of a 96-well plate containing either 100 mM Paraquat or 2.5 mM Sodium Arsenite, both diluted in M9 buffer. Worm death was determined by the absence of response to a gentle tap with a picker and survival was evaluated at intervals of 2 h [44].

### 2.4 Western blotting

Protein samples from *C. elegans* were quantified using the Bradford reagent subsequent to homogenisation in an alkylating lysis buffer. 20 μg of protein per each condition was applied to 12% reducing or non-reducing SDS PAGE gels. Protein transfer was peformed with a semi-dry blotter and the membrane underwent Ponceau S staining for normalisation. Following washing with TBS-T, the membranes were blocked in 5% milk within TBS-T for 1 h at room temperature. Following the blocking step, the membranes were exposed to primary antibodies (rabbit anti-Peroxiredoxin 2 and rabbit anti-Peroxiredoxin-SO_3_) at a dilution of 1:1000 in 5% milk incubated overnight. Subsequently, membranes were washed three times with TBS-T, each lasting 10 min. Membranes underwent incubation with the secondary antibody at a dilution of 1:10,000 in TBS-T, conducted in the dark, for a duration of 1 h. Image acquisition was performed using the Odyssey Fc imaging system (Li-Cor). The subsequent analysis of blot quantification and normalisation was performed employing Image Studio Lite.

### 2.5 qPCR

RNA isolation from *C. elegans* was conducted utilising TRIzol, following the methodology described in [45]. Following RNA isolation, cDNA synthesis and real-time qPCR were executed employing established protocols. For mRNA synthesis, 500 ng of RNA was mixed with 1 μl of random hexamers and incubated at 65 °C for 10 min. Then, the mixture was combined with a master mix including 4 μl of RT buffer, 2 μl of DTT, 1 μl of dNTP, 1 μl of Superscript II and 1 μl of Ribolock, followed by incubation at 42 °C for 60 min. The sequences of primers used in this study are listed in Table S2. Subsequent qPCR analysis was conducted using the SYBR Green Master Mix in a 10 μl reaction volume. The quantification of gene expression, relative to the housekeeping gene CDC42, was determined utilising the delta Ct method.

### 2.6 Imaging of *C. elegans*

A total of 30–45 worms per condition were subjected to the staining procedures immediately after the acute exercise or following a 24-h recovery period. The worms were incubated in 2.5 μM MitoTracker Red CMXRos for 10 min, 10 μM MitoSOX Red for 1 h, or 25 μM H2DCFDA for 1 h. After the probe incubation, the worms were transferred to NGM plates for 2–3 h in the dark room to avoid accumulation of the stain in the guts. Subsequently, the worms were immobilised with 20 mM levamisole and imaged using EVOS at 10× magnification [23].

To determine SKN-1 activation, the SKN-1 reporter CL2166 (*dvIs19 [(pAF15)gst-4p::gfp III*) strain was used, a cohort of 30–45 worms at the different ages underwent acute swimming. Post-exercise, the worms were immobilised on unseeded NGM plates for imaging using EVOS microscopy at a ×10 magnification. The assessment of green fluorescence intensity throughout the entire body of each worm was conducted using ImageJ [23].

The assessment of mitochondrial dynamics in the body wall muscle was performed with the SJ4103 strain (*zcIs14 [myo-3::gfp(mit)]*), using 30–45 worms subjected or not to acute swimming. Post-exercise, immobilised worms were placed on an agar slide with a coverslip. Subsequent imaging of mitochondria within the body wall muscles, specifically the region between the pharynx and vulva, was conducted under EVOS M7000 microscopy at a magnification of ×60. A total of 130–150 images were acquired and subsequently categorised into five distinct classes: Class 1, signifying highly abundant and well-networked mitochondria with minimal or no blebbing; Class 2, categorised by highly abundant mitochondria with network gaps and some blebbing; Class 3, featuring less abundant mitochondria with network gaps and increased blebbing; Class 4, indicating sparse mitochondria with some blebbing; and Class 5, denoting very sparse mitochondria [46].

To evaluate mitophagy, the IR2539 strain (*unc-119(ed3); Ex[pmyo-3 tomm-20::Rosella;unc-119(+)]*) was used. A group consisting of 30–45 worms underwent imaging at 60× magnification using the EVOS M7000 microscope. The ratio of green to red fluorescence intensity within a representative head region of each individual worm was subsequently determined using ImageJ [23].

The nuclear localisation of DAF-16 was assessed using the OH16024 strain (*daf-16(ot971[daf-16::GFP]) I.*) and TJ356 strain (*zIs356 [daf-16p::daf-16a/b::GFP + rol-6(su1006)]*). 30–45 worms were immobilised on an agar slide covered with a coverslip. Subsequent imaging of DAF-16 nuclear localisation within the body wall muscles, specifically the region between the pharynx and vulva, was conducted under EVOS M7000 microscopy at a magnification of ×60. A total of 130–150 images were acquired and subsequently categorised into three distinct classes: nuclear, where the DAF-16::GFP distribution was visible in the nuclear of body wall muscle; intermediate, where the DAF-16::GFP distribution was not completely visible, showing punctate fluorescence in the cytosol; and cytosolic, where the DAF-16::GFP distribution was in the cytosol [18].

### 2.7 Transmission electron microscopy

*C. elegans* strains were fixed with osmium tetroxide. Subsequently, the specimens underwent a dehydration process through a graded series of ethanol concentrations (30%, 50%, 70%, 90% and finally 100%), with each step lasting 2 × 15 min. Following the last 100% ethanol dehydration step, acetone was used to replace ethanol in a 2 × 20-min procedure. Subsequently, the samples were immersed in a 50:50 mixture of resin and acetone for 4 h, followed by placement in a 75:25 mixture on a rotator overnight. The following day, samples were transferred into 100% resin and rotated for 5–6 h. Upon completion, the specimens were placed into the appropriate embedding mould, filled with fresh 100% resin and then subjected to an oven at 65 °C for 48 h for polymerisation. Finally, the moulded samples were sectioned to prepare them for imaging. 10 captured images with at least 30–45 mitochondria were acquired at a magnification of 25,000×. The ER was defined in the EM images by its characteristic profile, consisting of a network of interconnected membranous tubules and flattened sacs, studded with ribosomes. ImageJ was used according to [47] to quantify, mitochondrial length (mitochondrial longitudinal distance), mitochondrial width (mitochondrial lateral distance), aspect ratio (ratio of mitochondrial length/width), mitochondrial area (mitochondrial area size), MERC length (contacted length between mitochondria and ER), MERC distance (contacted distance between mitochondria and ER) and ERMICC (ratio of MERC length to product of mitochondrial perimeter and MERC distance),.

### 2.8 CeleST

To evaluate the swimming proficiency of *C. elegans*, CeleST analysis was performed following the acute exercise and the revery period. A minimum of 30 worms, arranged in 4–5 individuals per trial, were positioned on a glass substrate within a 10 mm ring. Subsequently, 60-s video recordings were captured at a rate of 16 frames per second, utilizing a Nikon LV-TV microscope set at 1× magnification and equipped with an OPTIKA C-P20CM camera [23,48].

### 2.9 Statistical analysis

Images obtained from Western blot and microscopy of *C. elegans* were subjected to semi-automated quantification using ImageJ and Image Studio Lite and manual corrections were applied as needed to ensure accuracy in the quantification process. For the MitoTracker, MitoSOX, DCFDA and CL2166 strain staining, the quantification involved a total of 30–45 worms, with the fluorescence intensity measured per worm for each specific experimental condition. For SJ4103 strain, an evaluation was conducted on 130–150 images captured in the region between the pharynx and vulva and assessment was carried out based on the five predefined categories described earlier. For TJ356 and OH16024 strains, 130–150 images of body wall muscle were captured and quantified based on the DAF-16::GFP distribution categorised as nuclear, intermediate and cytosolic. For the IR2539 strain, the assessment involved determining the ratio of green fluorescence to red fluorescence for each individual worm. The specific details regarding the statistical analyses conducted are provided in the respective figure legends. To assess differences between two groups, a Student's t-test was employed. For comparisons involving more than two groups, either one-way or two-way ANOVA was utilised. The log-rank test was employed for comparing tolerance in the oxidative stress assay. The distribution into multiple classes was analysed using the chi-square test. A significance threshold of *p*-value <0.05 was considered statistically significant. All graphical representations and statistical analyses were performed using GraphPad Prism 7. The source data used for figure generation in this manuscript are available in the source data file.

## 3 Results

### 3.1 Redox mediated adaptation to exercise diminishes with age through a PRDX-2 dependent pathway

**Figure 1:**
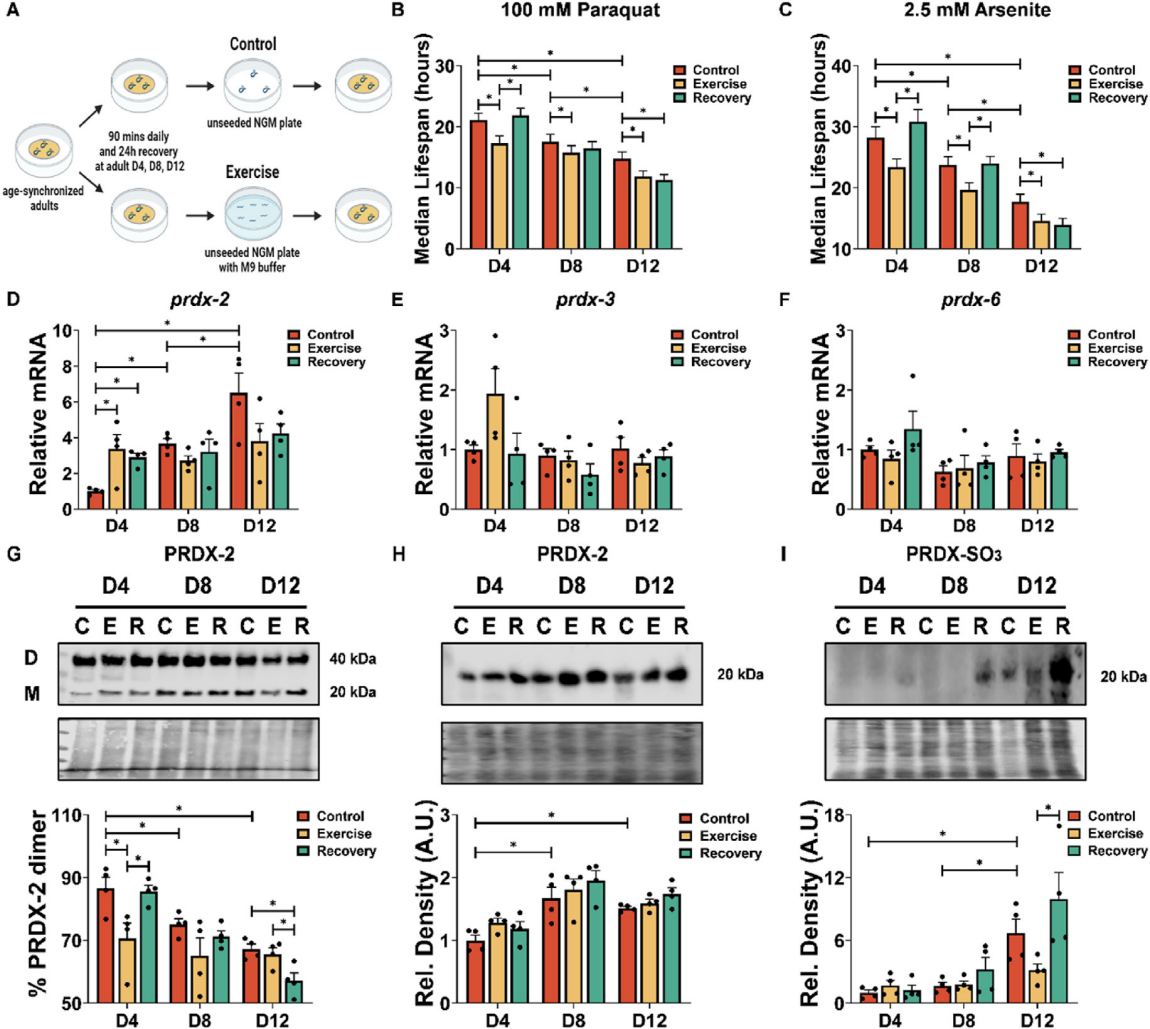
**Decreased survival with exposure to oxidative stress, alterations in Peroxiredoxin mRNA and PRDX-2 in response to ageing and exercise.** (A) Schematic of acute swimming protocol for *C. elegans* at different ages. (B and C) Survival to paraquat and arsenite following acute exercise as well as ageing (n = 60). (D–F) mRNA levels of *prdx-2*, *prdx-3* and *prdx-6* following acute exercise and ageing (n = 4). (G–I) Percentage dimer/total (dimer + monomer) ratio of PRDX-2 (G), protein levels of PRDX-2 (H) and PRDX-SO_2_/SO_3_ (I) (n = 4). Graphs are the normalised relative means ± SEM and *p*-value of <0.05 was considered as statistically significant ∗(*p* < 0.05). (B: D4: C vs E = 0.0034, E vs R = 0.001; D8: C vs E = 0.0435; D12: C vs E = 0.022, C vs R = 0.0076; D4 vs D8: = 0.0266; D4 vs D12 < 0.0001; D8 vs D12 = 0.0181. C: D4: C vs E = 0.0114, E vs R = 0.0009; D8: C vs E = 0.0143, E vs R = 0.0079; D12: C vs E = 0.031; C vs R = 0.0048; D4 vs D8: = 0.0136; D4 vs D12 < 0.0001; D8 vs D12 = 0.0023. D: D4: C vs E = 0.017, C vs R = 0.0485; D4 vs D8 = 0.0445; D4 vs D12 = 0.0006. G: D4: C vs E = 0.0325; E vs R = 0.0432; D12: C vs R = 0.0173, E vs R = 0.0402; D4 vs D8 = 0.0237; D4 vs D12 = 0.0009. H: D4 vs D8 = 0.0063; D4 vs D12 = 0.0271. I: D12: E vs R = 0.0475; D4 vs D12 = 0.002; D8 vs D12 = 0.0044).

To explore the adaptive response to ageing and exercise, we employed *C. elegans*, a model organism that has been validated as an effective platform for studying physiological responses to exercise [23,46,48]. A 90-min swimming exercise has been demonstrated to elicit muscle fat consumption, increase locomotory fatigue and elevate mitochondrial ROS levels [43]. Therefore, we conducted a single 90-min swim exercise session at distinct ages, adult (D4), late middle age (D8) and old (D12) worms (Figure 1A) in N2 wild type strain. Initially, worms were subjected to diverse forms of oxidative stress, including exposure to paraquat (PQ) and arsenite (AS) following an exercise regimen and subsequent recovery. A discernible reduction in the adaptive response capacity was observed with progressive ageing. The resistance to both PQ and AS decreased immediately after acute exercise (Figure 1B,C). Subsequently, a recovery was observed 24 h post-exercise in the younger D4 and D8 worms, contrasting with the absence of a recovery in D12 worms (Figure S1A–H). The mean lifespan of each condition is detailed in Table S1. These results demonstrate a decreased capacity to withstand stress with age and older worms are not able to recover following a physiological exercise stress.

Given the previously demonstrated involvement of PRDX-2 in the redox-mediated adaptation to chronic exercise in our prior investigations [23], we conducted qPCR and Western blot analyses following a regimen of acute exercise and subsequent recovery. Initially, we examined the mRNA expression levels of the Peroxiredoxin genes in *C. elegans*, encompassing *prdx-2*, *prdx-3* and *prdx-6*, in response to the exercise and recovery protocol. Intriguingly, our observations unveiled a distinctive sensitivity pattern, with only *prdx-2* exhibiting an adaptive response to both acute exercise ageing. Specifically, following acute exercise and post-exercise in D4 worms, *prdx-2* but not *prdx-3* or *prdx-6* demonstrated an elevated expression level (Figure 1D–F). There was no significant change of *prdx-2* expression in D8 and D12 worms in response to exercise. Furthermore, a gradual increase in the expression of *prdx-2* was observed in control conditions at D8 and D12 (Figure 1D). The ratio of PRDX-2 Dimer:Monomer was analysed using non-reducing gel electrophoresis. Exercise resulted in a pronounced shift in the dimerisation of PRDX-2 in D4 worms but returned to control levels following a recovery period (Figure 1G). Although gene expression of *prdx-2* increased following exercise in D4 worms there was no significant change in the abundance of PRDX-2. D8 and D12 worms had increased abundance of PRDX-2 but decreased % PRDX-2 dimer formation compared to D4 worms (Figure 1G,H). There was also an increase in hyperoxidised Peroxiredoxins in D12 worms compared to D4 and D8 worms, which increased further following the recovery period (Figure 1I). The shift in the redox state of PRDX-2 to a monomer following acute ROS generation could be reflective of an increase in PRDX-2 hyperoxidation, which can inhibit the formation of disulphide bonds and dimer formation. These findings demonstrate that resistance to oxidative stress declines with ageing, and exercise was associated with distinct changes in the sensitivity of the redox state of PRDX-2 to exercise and age.

### 3.2 PRDX-2 is required for mitochondrial adaptations in response to exercise

**Figure 2:**
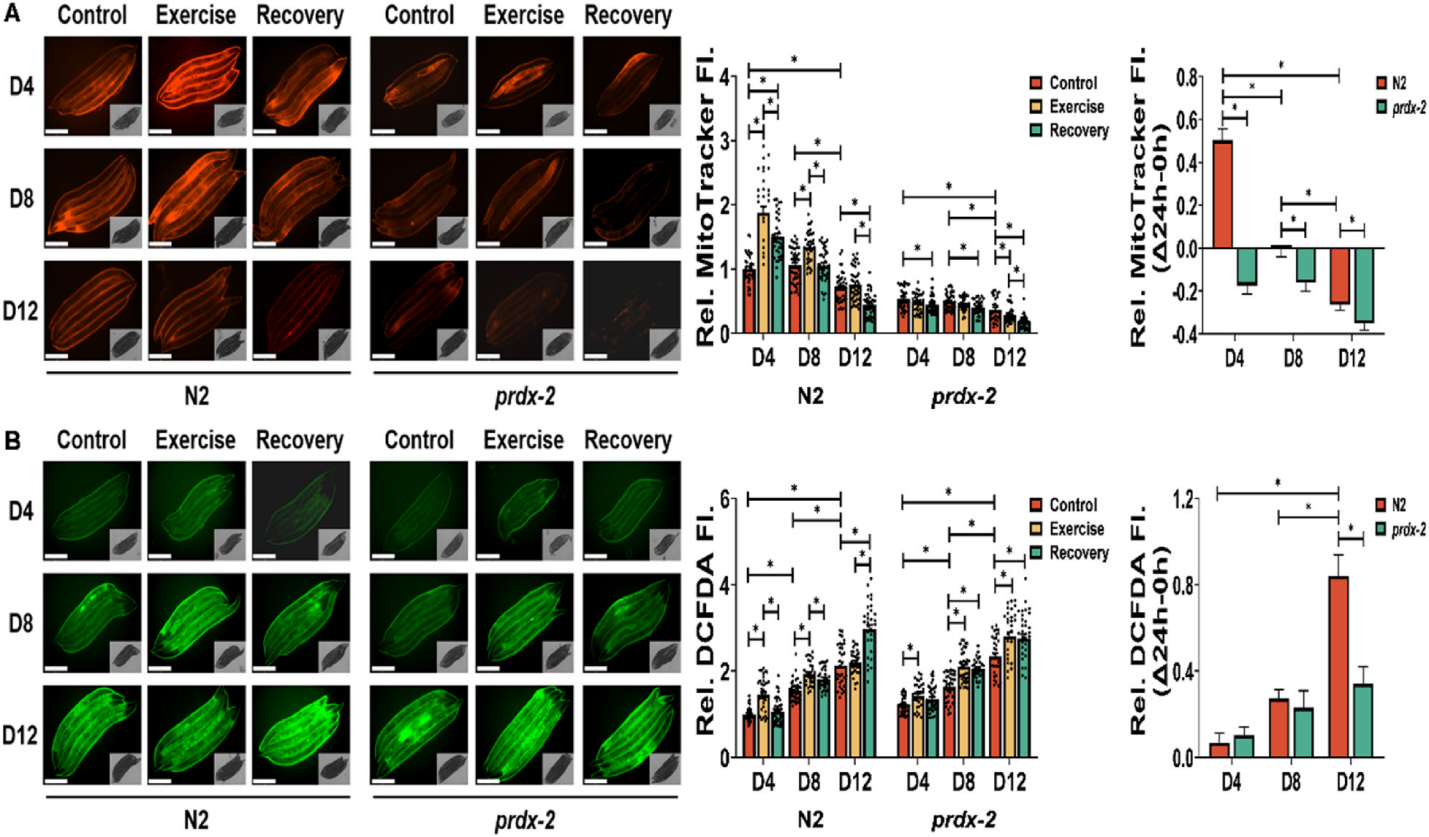
**Ageing and loss of PRDX-2 result in decreased membrane potential and increased ROS resulting in disrupted adaptation to exercise.** (A–B) MitoTracker red staining as an indicator of mitochondrial membrane potential (A) and DCFDA staining for intracellular ROS (B) following acute exercise at different stages, scale bar = 275 μm, n = 30–40. Graphs are the normalised relative means ± SEM and *p*-value of <0.05 was considered as statistically significant ∗(*p* < 0.05). *p* values (A: in N2 worms: D4: C vs E < 0.0001, C vs R < 0.0001, E vs R = 0.0002; D8: C vs E < 0.0001, E vs R < 0.0001; D12: C vs R < 0.0001, E vs R < 0.0001; D4 vs D12 < 0.0001; D8 vs D12 < 0.0001; in *prdx-2* worms: D4: C vs R = 0.0183; D8: C vs R = 0.0079; D12: C vs E = 0.0361, C vs R < 0.0001, T vs R = 0.0086; D4 vs D12 = 0.0001; D8 vs D12 = 0.005; recovery rate: N2: D4 vs D8 = 0.0035; D4 vs D12 = 0.0007; *prdx-2*: D4 vs D12 = 0.002; D8 vs D12 = 0.0022; N2 vs *prdx-2*: D4 < 0.0001; D8 = 0.0067; D12 = 0.0295. B: in N2 worms: D4: C vs R < 0.0001, E vs R < 0.0001; D8: C vs E < 0.0001, C vs R < 0.0001; D12: C vs R < 0.0001; E vs R < 0.0001; D4 vs D8 < 0.0001; D4 vs D12 < 0.0001; D8 vs D12 < 0.0001. In *prdx-2* worms: D4: C vs E = 0.005; D8: C vs E < 0.0001, C vs R < 0.0001; D12: C vs E = 0.001, C vs R = 0.0035; D4 vs D8 < 0.0001; D4 vs D12 < 0.0001; D8 vs D12 < 0.0001. Recovery rate: N2: D4 vs D12 < 0.0001; D8 vs D12 < 0.0001; *prdx-2*: D4 vs D12 = 0.0297; N2 vs *prdx-2*: D12 = 0.0002).

In order to determine the role of PRDX-2 in mitochondrial adaptations in response to exercise, we performed MitoTracker, MitoSOX and DCFDA staining using N2 and *prdx-2* (VE1) mutant strains throughout a structured exercise and recovery regimen at different ages. A progressive reduction in MitoTracker Red staining intensity, indicative of mitochondrial membrane potential, was noted at D8 and D12 worms relative to D4 worms control groups in both N2 and *prdx-2* mutant strains (Figure 2A). Subsequent analysis revealed an immediate increase in staining intensity and mitochondrial membrane potential post-acute swim, with recovery to control levels observed 24 h after exercise in D4 and D8 worms in the N2 strain. This trend was absent in the old D12 N2 strain. MitoTracker red staining was decreased in the *prdx-2* mutant strain and unlike the N2 strain, there was no increase in staining at any age following the exercise protocol. Notably, a gradual decline in the recovery rate (Δ24 h–0 h) was observed with age in both N2 and *prdx-2* mutant strains, with N2 consistently exhibiting a superior recovery rate compared to the *prdx-2* mutant strain across all ages (Figure 2A). To assess the adaptive response to exercise-induced ROS, we utilised DCFDA staining as a proxy for overall cellular ROS and MitoSOX staining as an indicator for mitochondrial ROS. We observed an elevation in DCFDA and MitoSOX intensity at D4 and D8 post-exercise in both N2 and *prdx-2* mutant strains (Figures 2B and S2A). However, this increase was transient in the N2 strain and returned to basal levels following the recovery period but remained elevated in the *prdx-2* mutant strain at D4 and D8. There was a progressive escalation of DCFDA and MitoSOX staining intensity with advancing age compared to D4 worms in both strains. Remarkably, in old worms at D12, an immediate increase of DCFDA and MitoSOX staining intensity was not observed directly after exercise, instead a significant increase was noted 24 h later (Figures 2B and S2A). The *prdx-2* mutant strain was crossed with a reporter for SKN-1 activation, CL2166 *dvIs19[(pAF15)gst-4p::GFP::NLS] III* to assess SKN-1 (ortholog of NRF2) activation during both exercise and ageing process. The analysis revealed a gradual elevation of SKN-1 activity at D8 and D12 in both wild type and *prdx-2* mutant strains (Figure S2B). Furthermore, there was an increase in SKN-1 activity 24 h post-acute exercise at D4, although this effect was not observed at D8 or D12 in the N2 strain. Intriguingly, a decrease in SKN-1 activity was noted during the recovery phase in *prdx-2* mutants at D8 and D12. Consistent with MitoTracker staining results, the SKN-1 recovery rate exhibited a decline with ageing in both wild type and *prdx-2* mutant strains, the wild type strain demonstrated a superior recovery rate compared to the *prdx-2* mutant throughout the lifespan (Figure S2B). Collectively, the data indicate an acute increase in ROS and mitochondrial membrane potential following exercise in the N2 strain that returns to baseline levels following a recovery in D4 and D8 worms. There was a gradual increase in overall ROS with age and a decline in mitochondrial membrane potential. The increase in ROS corresponds to the oxidative shift in the redox state of PRDX-2 demonstrated in Figure 1G,I. The *prdx-2* mutant strain had reduced mitochondrial membrane potential and exercise induced an increase in ROS at D4 and D8, which did not return to baseline following a recovery period. The results highlight the essential role of PRDX-2 in mediating the adaptive response to exercise.

### 3.3 Loss of PRDX-2 results in fragmented mitochondria and disrupted mitochondrial dynamics

**Figure 3:**
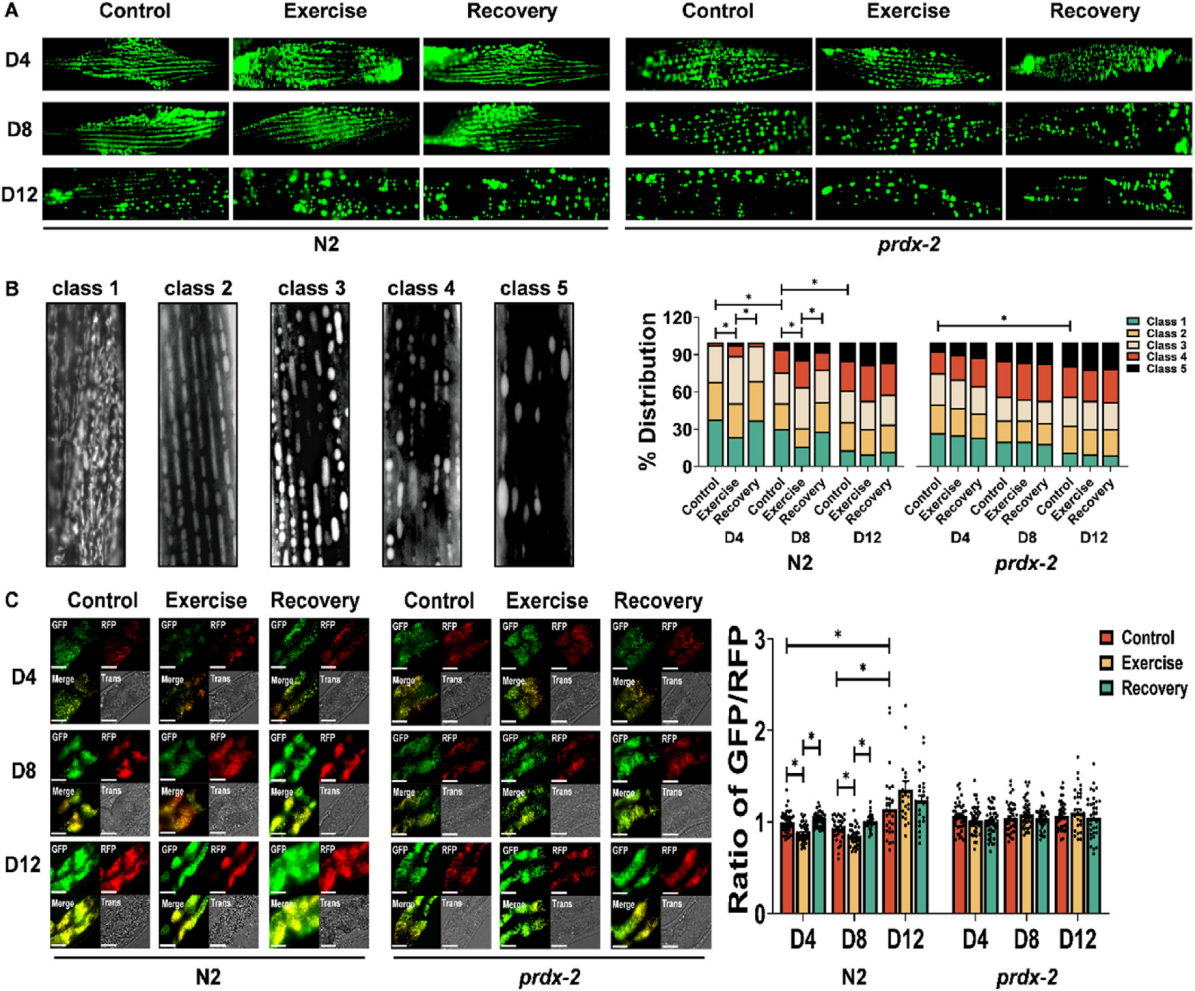
**Ageing and loss of PRDX-2 results in mitochondrial fragmentation and disrupted mitophagy.** (A–C) Representative images of *myo-3::gfp* reporter strain for mitochondrial morphology (A, B) and *myo3-p::tomm20::Rosella* reporter strain for mitophagy (C) following acute exercise at different ages, scale bar = 50 μm, n = 130–150 for mitochondria reporter, n = 30–45 for mitophagy reporter. Graphs are the normalised relative means ± SEM and all experiments were performed and *p*-value of <0.05 was considered as statistically significant ∗(*p* < 0.05). *p* values (B: in N2 worms: D4: C vs E = 0.03, E vs R = 0.0456; D8: C vs E = 0.041, E vs R = 0.0478; D4 vs D8 = 0.0004; D8 vs D12 = 0.0007; in *prdx-2* worms: D4 vs D12 = 0.0135. C: in N2 worms: C vs E < 0.0001, E vs R < 0.0001; D8: C vs E = 0.0063; E vs R < 0.0001; D4 vs D12 = 0.0194; D8 vs D12 = 0.0004).

To elucidate the impact of PRDX-2 on mitochondria in response to exercise, the *prdx-2* mutant strain was crossed with the muscle mitochondrial reporter strain SJ4103 *zcIs14 [myo-3::GFP(mit)]* for monitoring mitochondrial morphology and IR2539 *unc-119(ed3); Ex[pmyo-3TOMM-20::Rosella;unc-119(+)]* to determine mitophagy. Mitochondrial morphology was quantified within the body-wall muscle of *C. elegans* (Figure 3A), a tissue displaying numerous parallels to mammalian skeletal muscle [49]. This analysis entailed the classification of mitochondrial morphology into five distinct categories, each indicative of a stepwise escalation in the levels of mitochondrial fragmentation and disorganisation (Figure 3B). The majority of body wall muscles exhibited plentiful and interconnected mitochondria in D4 N2 worms. At D8 and D12, there was an increase in mitochondrial fragmentation and progressive impairment in mitochondrial connectivity (Figure 3A). Furthermore, acute exercise induced mitochondrial fragmentation in body wall muscles, with subsequent restoration of filamentous mitochondria observed 24 h post-exercise in D4 and D8 worms, but not in D12 worms (Figure 3B). The *prdx-2* mutant strain exhibits an accelerated ageing phenotype [23,50] and a highly fragmented mitochondrial network was apparent at D4 that further deteriorates with age, suggesting a critical role for PRDX-2 in preserving mitochondrial integrity during both acute exercise and the ageing process. The evaluation of mitochondrial turnover involved the use of a mitophagy reporter strain *[myo-3p tomm-20::Rosella]*. The Rosella biosensor consists of a pH-stable RFP fused with a pH-sensitive GFP. Under basal conditions, the mitochondrial network displays red and green fluorescence. During mitophagy, as mitochondria undergo transportation to the acidic lysosomal environment, the GFP fluorescence undergoes quenching, while the RFP fluorescence remains stable [51]. The GFP/RFP ratio was quantified to assess mitophagy [23]. A heightened ratio, indicative of decreased mitophagy, was apparent in D12 worms compared to D4 worms. Interestingly, the 90-min swim regimen induced mitophagy and recovery was evident 24 h later in D4 and D8 worms, whereas D12 worms did not exhibit a change in mitophagy (Figure 3C). The obvious alterations of GFP/RFP during ageing and exercise, were not observed in *prdx-2* mutant strain at any age (Figure 3C). These findings demonstrate that ageing results in an increase in mitochondrial fragmentation and decreased mitophagy. Exercise promoted mitochondrial fragmentation and mitophagy but the recovery was inhibited with age. The data highlight that loss of PRDX-2 resulted in increased mitochondrial fragmentation and potentially disrupted mitochondrial fusion along with a failure to activate mitophagy with exercise.

### 3.4 Exercise induces DAF-16 nuclear localisation and promotes an acute increase in genes regulating mitochondrial morphology that is absent in the *prdx-2* mutant strain

**Figure 4:**
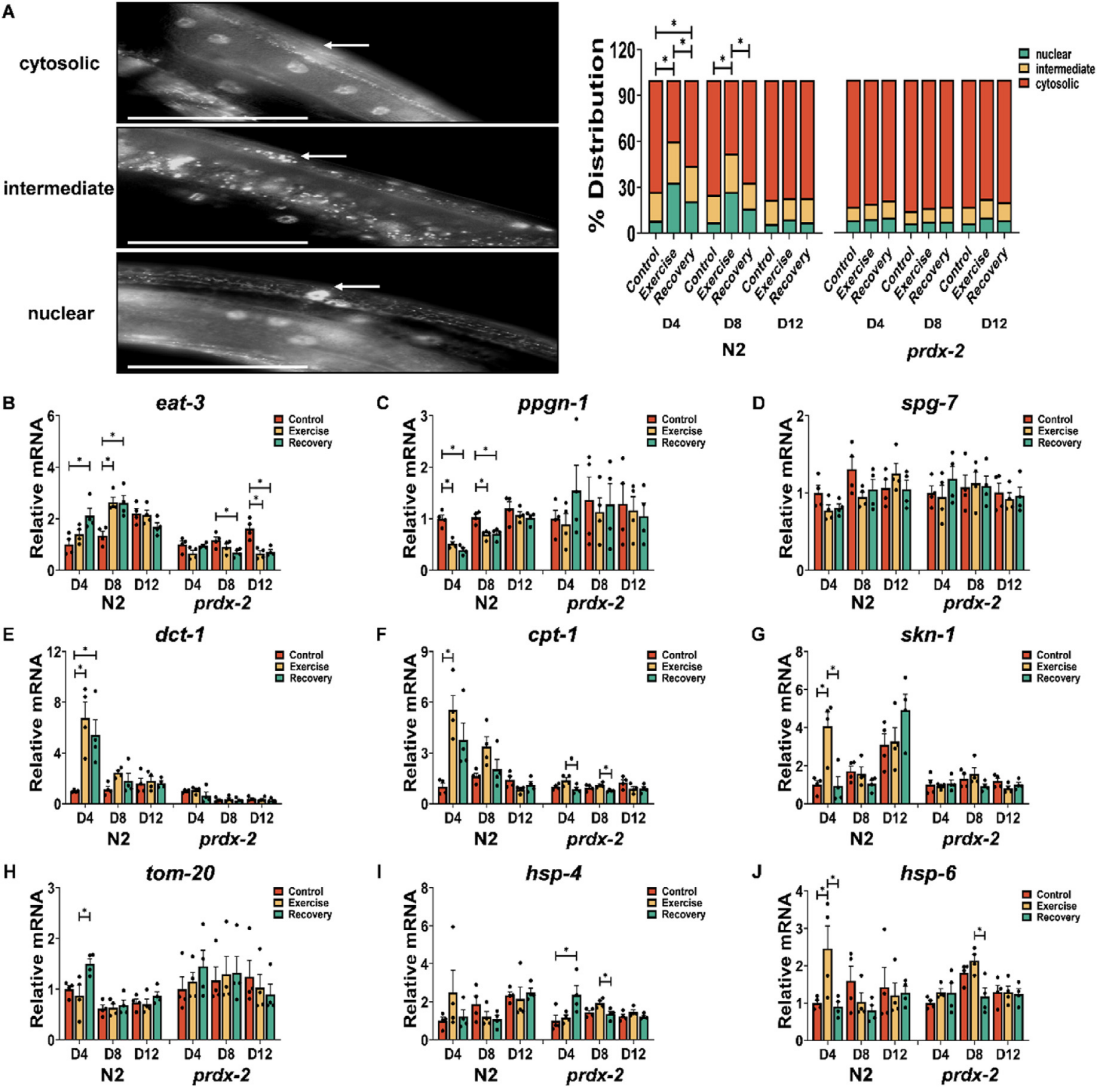
**Ageing and loss of PRDX-2 adversely impacts the DAF-16 nuclear localisation and the expression of genes regulating mitochondrial dynamics and UPR in response to exercise.** (A) Representative images of the OH16024 strain DAF-16::GFP distribution, scale bar = 50 μm, n = 130–150. (B–J) mRNA level of *eat-3* (B), *ppgn-1* (C), *spg-1* (D), *dct-1* (E), *cpt-1* (F), *skn-1* (G), *tom-20* (H), *hsp-4* (I) and *hsp-6* (J) following acute exercise at different stages, n = 4. Graphs are the normalised relative means ± SEM and all experiments were performed with n = 130–150 for DAF-16 reporter, n = 4 for mRNA expression and *p*-value of <0.05 was considered as statistically significant ∗(*p* < 0.05). *p* values (A: in N2 worms: D4: C vs E < 0.0001, E vs R = 0.046, C vs R = 0.0182; D8: C vs E < 0.0001, E vs R = 0.0238. B: in N2 worms: D4: C vs R = 0.0157, D8: C vs E = 0.0059, C vs R = 0.0066; in *prdx-2* worms: D8: C vs R = 0.0409; D12: C vs E = 0.0019, C vs R = 0.0029. C: in N2 worms: D4: C vs E = 0.0004, C vs R < 0.0001; D8: C vs E = 0.0034, C vs R = 0.005. E: in N2 worms: D4: C vs E = 0.0073, C vs R = 0.0305. F: in N2 worms: D4: C vs E = 0.0063. G: in N2 worms: D4: C vs E = 0.0062, E vs R = 0.0055. H: in N2 worms: D4: E vs R = 0.0242. I: in *prdx-2* worms: D4: C vs R = 0.0467; D8: E vs R = 0.0258. J: in N2 worms: D4: C vs E = 0.0449, E vs R = 0.0337; in *prdx-2* worms: D8: E vs R = 0.0108).

To elucidate the molecular consequences of alterations in mitochondrial adaptations during exercise and the ageing process, the *prdx-2* mutant strain was crossed with DAF-16 reporter strains OH16024 *daf-16(ot971[daf-16::GFP]) I* and TJ356 *zIs356 [daf-16p::daf-16a/b::GFP + rol-6(su1006)]*. Moreover, mRNA levels of genes associated with mitochondrial morphology were quantified in both the N2 and *prdx-2* mutant strains. The distribution of DAF-16::GFP was quantified within the body-wall muscle of *C. elegans*, specified into three categories: cytosolic, intermediate and nuclear (Figure 4A). The acute exercise induced DAF-16 nuclear localisation in D4 and D8 worms, but not in D12 worms (Figures 4A and S3A). The *prdx-2* mutant did not induce DAF-16 nuclear localisation following exercise at any age. Previous studies have demonstrated that *prdx-2* mutants at L4 stage have elevated resistance to sodium arsenite, as a result of increased DAF-16 nuclear localisation [52]. We confirmed these results at the L4 stage but at D4 there was decreased resistance to sodium arsenite and DAF-16 nuclear localisation (Figure S3B and C). Inhibition of *spg-7* and *ppgn-1* expression by DAF-16 nuclear localisation, alleviates the negative regulation of mitochondrial fusion protein EAT-3 by mitochondrial proteases SPG-7 and PPGN-1 [18]. There was decreased expression of *ppgn-1* and increased expression of *eat-3* in N2 at adult D4 and adult D8 following exercise, but not in the *prdx-2* mutant (Figure 4B–D). Furthermore, the expression levels of *dct-1* (ortholog of BNIP3 mitophagy receptor), *cpt-1* (ortholog of CPT1, a marker of long chain fatty acid oxidation and regulator of mitochondrial morphology) and *skn-1* exhibited a notable increase either immediately or 24 h post-exercise, particularly in D4 worms (Figure 4E–G). In contrast, D12 worms did not display a similar elevation in the expression of any of these genes following exercise. Notably, the *prdx-2* mutant strain did not demonstrate an observable upregulation of these genes (*dct-1*, *cpt-1* and *skn-1*) following exercise at any age (Figure 4E–G). Moreover, it was observed that the expression levels of *eat-3* and *skn-1* exhibit a gradual increase with advancing age (Figure S3D and I). The results demonstrate that exercise induced DAF-16 nuclear localisation and expression of genes promoting mitochondrial remodelling, these changes were absent in old worms and the *prdx-2* mutant strain.

### 3.5 PRDX-2 is required for exercise induced UPR activation and mitochondrial ER remodelling

**Figure 5:**
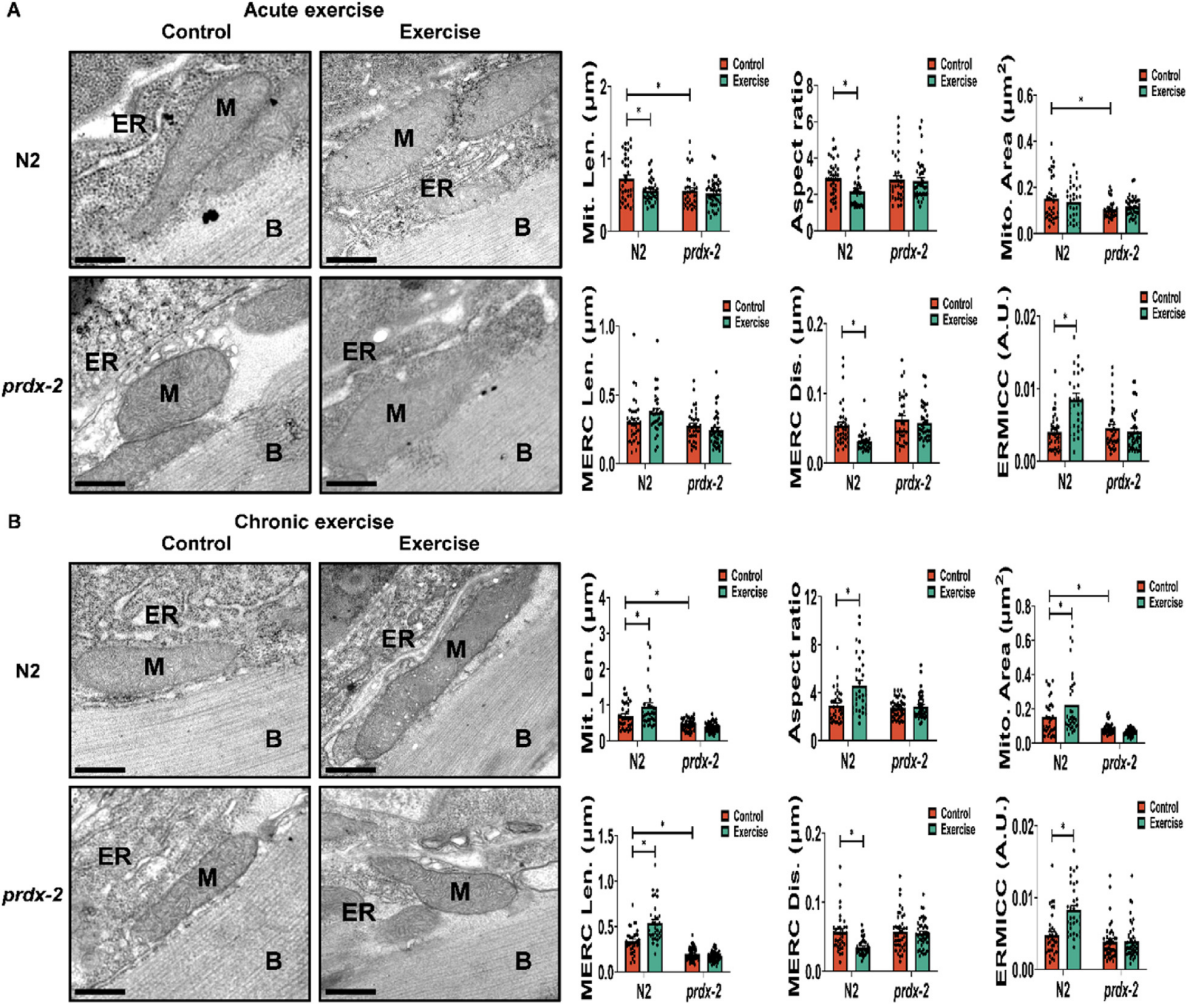
**Exercise influences mitochondrial ER contact sites in N2 but not in the *prdx-2* mutant.** (A–B) Mitochondria ER contact sites change following acute exercise (A) and chronic exercise (B), scale bar = 200 nm, n = 30–45. Graphs are the normalised relative means ± SEM and all experiments were performed and *p*-value of <0.05 was considered as statistically significant ∗(*p* < 0.05). *p* values (A: mitochondrial length: N2: C vs E = 0.0231, N2 vs *prdx-2* = 0.0184. Aspect ratio: N2: C vs E = 0.0317. Mitochondrial area: N2 vs *prdx-2* = 0.0126. MERC distance: N2: C vs E = 0.0046. ERMICC: N2: C vs E < 0.0001. B: mitochondrial length: N2: C vs E = 0.0142; N2 vs *prdx-2* = 0.0162. Aspect ratio: N2: C vs E < 0.0001. Mitochondrial area: N2:C vs E = 0.0203; N2 vs *prdx-2* = 0.0225. MERC length: N2: C vs E < 0.0001; N2 vs *prdx-2* < 0.0001. MERC distance: N2:C vs E = 0.001. ERMICC: N2:C vs E < 0.0001).

Mitochondria and the ER are essential regulatory centres in cellular homeostasis, engaging in a synergistic relationship. Communication between these organelles is facilitated by MERCS, which have been identified as key regulators of both mitochondrial fission and fusion [24,25,28]. The activation of the adaptive Unfolded Protein Response (UPR) is linked with remodelling of ER and MERCS [26]. Expression of *hsp-4* (reflection of ER-associated UPR) and *hsp-6* (an indication of mitochondrial UPR) was quantified following exercise and during the ageing process. Results indicated a heightened expression of *hsp-6* following exercise at D4 in the N2 strain (Figure 4J). Furthermore, the expression levels of *hsp-4* increased at D8 and D12 compared to D4 in the N2 strain (Figure S3K). Notably, the *prdx-2* mutant strain did not demonstrate an upregulation of *tom-20* and *hsp-6* following exercise at any age (Figure 4H,J). The link between activation of the UPR following ER stress has been suggested to regulate MERCS formation [53]. Transmission electron microscopy (TEM) imaging of mitochondria and ER was employed to quantify mitochondrial morphology and MERCS formation in response to the cellular adaptations to exercise [47]. In N2 worms, the acute exercise induced a reduction in mitochondrial length and aspect ratio (length/width), implying a transition in muscle mitochondrial morphology from rod-shaped to oval-shaped, but this alteration was not observed in the *prdx-2* mutant strain (Figure 5A). Following 5 days of exercise, there was a notable increase observed in mitochondrial length, area and aspect ratio in the N2 strain (Figure 5B). These findings suggest an augmentation in mitochondrial mass as a consequence of the chronic exercise regimen. In the comparison between adult D4 and D8 worms, it was observed that *prdx-2* mutants exhibited a more fragmented mitochondria relative to N2 worms, consistent with the highly fragmented mitochondrial network observed in the *prdx-2* mutant strain (Figure 3A). Notably, both acute and chronic exercise interventions resulted in a significant reduction in MERC distance, representing a closer coupling between mitochondria and ER in the N2 strain. Furthermore, a notable increase in the frequency of Endoplasmic Reticulum-Mitochondria Contact Compartments (ERMICCs: calculated as MERC length divided by the product of mitochondrial perimeter and MERC distance) [54] was observed following both acute and chronic exercise interventions in the N2 strain but not in the *prdx-2* mutant strain (Figure 5A,B). These findings substantiate the presence of a substantial and dynamic interplay between mitochondria and the ER in response to exercise. Together, these findings suggest that PRDX-2 plays a key role in regulating UPR activation and MERCS formation following exercise.

### 3.6 PRDX-2 is required for increased fitness in response to exercise

**Figure 6:**
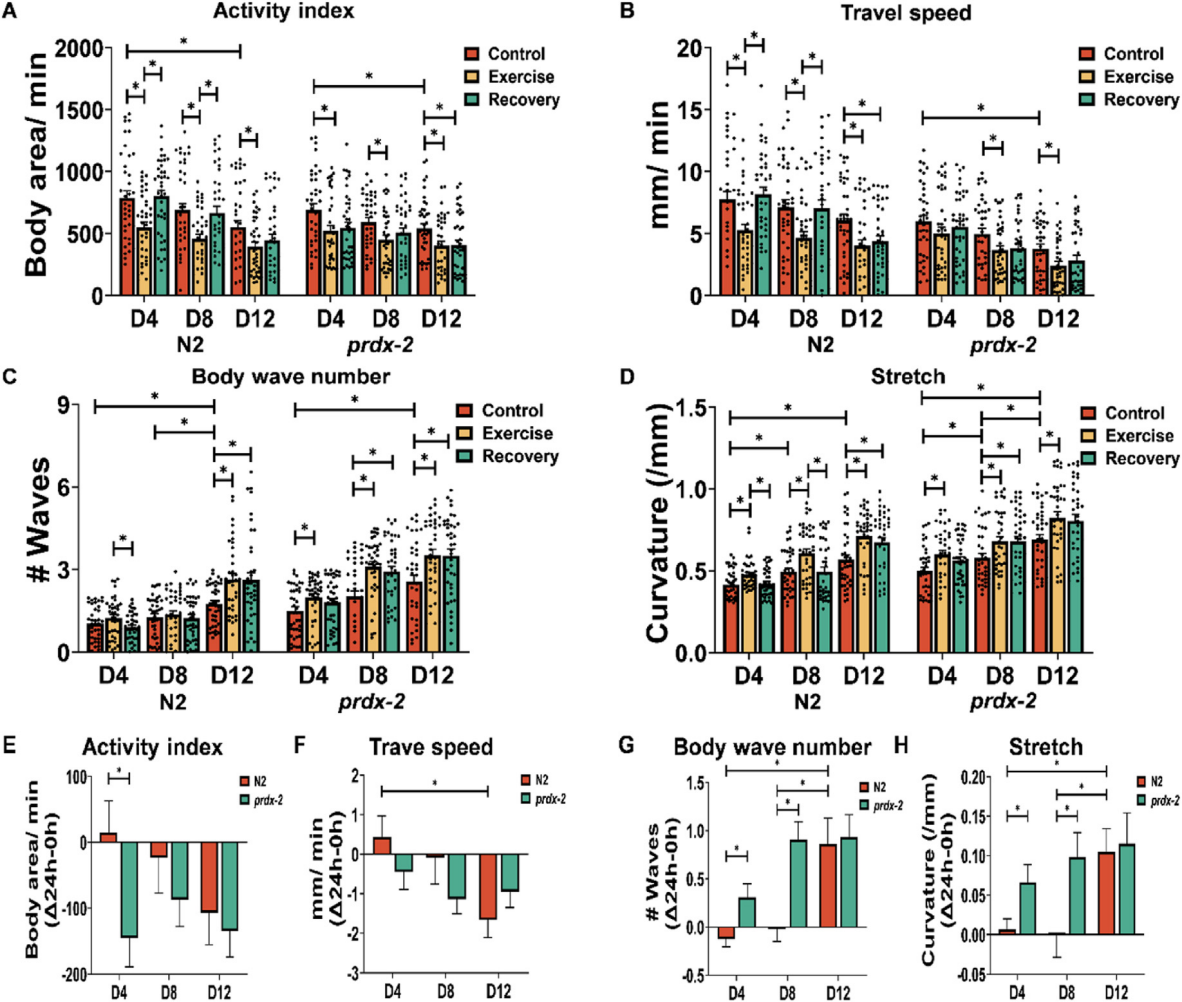
**Ageing results in a decline in physical fitness and *prdx-2* mutants cannot recover following an exercise intervention.** (A–D) Activity index (A), travel speed (B), body wave number (C) and stretch (D) following acute exercise at different stages, n = 30–40. (E–H) The recovery rate of activity index (E), travel speed (F), body wave number (G) and stretch (H) compared to normal condition, n = 30–40. Graphs are the normalised relative means ± SEM and *p*-value of <0.05 was considered as statistically significant ∗(*p* < 0.05). *p* values (A: in N2 worms: D4: C vs E = 0.0032, E vs R = 0.0017; D8: C vs E = 0.0027, E vs R = 0.0088; D12: C vs E = 0.0405; D4 vs D12 = 0.0073; in *prdx-2* worms: D4: C vs E = 0.0262; D8: C vs E = 0.0231; D12: C vs E = 0.0291, C vs R = 0.0362; D4 vs D12 = 0.0289. B: in N2 worms: D4: C vs E = 0.006, E vs R = 0.001; D8: C vs E = 0.0098, E vs R = 0.0148; D12: C vs E = 0.008, C vs R = 0.0346. In *prdx-2* worms: D8: C vs E = 0.0441; D12: C vs E = 0.0263; D4 vs D12 = 0.0008. C: in N2 worms: D4: E vs R = 0.0365; D12: C vs E = 0.0084; C vs R = 0.0097; D4 vs D12 < 0.0001; D8 vs D12 = 0.0058; in *prdx-2* worms: D4: C vs E = 0.0394; D8: C vs E = 0.0002, C vs R = 0.0022; D12: C vs E = 0.0091, C vs R = 0.01; D4 vs D12 = 0.0003. D: in N2 worms: D4: C vs E = 0.0067, E vs R = 0.0177; D8: C vs E = 0.0064, E vs R = 0.0069; D12: C vs E = 0.0016, C vs R = 0.0267; D4 vs D8 = 0.036; D4 vs D12 < 0.0001; in *prdx-2* worms: D4: C vs E = 0.0098; D8: C vs E = 0.0467, C vs R = 0.0479; D12: C vs E = 0.0279; D4 vs D12 < 0.0001; D8 vs D12 = 0.0153; E: N2 vs *prdx-2*: D4 = 0.0165. F: in N2 worms: D4 vs D12 = 0.0217. G: in N2 worms: D4 vs D12 = 0.0003; D8 vs D12 = 0.0018; N2 vs *prdx-2*: D4 = 0.009; D8 = 0.0001. H: in N2 worms: D4 vs D12 = 0.0151; D8 vs D12 = 0.0095; N2 vs *prdx-2*: D4 = 0.0268; D8 = 0.00221).

To investigate the physiological functional implications of exercise and ageing in both N2 wild-type strain and the *prdx-2* mutant strain that has an altered mitochondrial response capacity. CeleST [55] was implemented to quantify the activity patterns in these strains throughout the cycles of exercise and recovery at various ages. Parameters quantified included activity index, wave initiation rate, travel speed and brush stroke that are indicative of beneficial physical fitness, whereas body wave number, asymmetry, stretch and curling represent frailty [48]. In both strains, a consistent decline was observed in the activity index, travel speed, wave initiation rate and brush stroke with advancing age (Figures 6A, B, S4A and S4B). There was also an increase in body wave number, stretch, asymmetry and curling, exhibiting more pronounced effects in older worms, particularly at the D12 age (Figures 6C, D, S4C and S4D). Moreover, acute exercise induced alterations in these parameters, reverting to baseline in D4 and D8 worms following exercise, but not in old D12 worms. Importantly the *prdx-2* mutant strain did not recover following the exercise intervention at any age. In the comparative analysis of recovery rates in N2 at different ages, D4 worms exhibited superior recovery rates in travel speed, body wave number, stretch and wave initiation rate compared to D12 worms (Figures 6F–H and S4E). Additionally, N2 demonstrated a more robust recovery rate in activity index, body wave number, stretch and wave initiation rate than the *prdx-2* mutant, particularly notable at the D4 stage (Figures 6E, G, 6H and S4E). Furthermore, comprehensive quantification of these parameters at different ages revealed that the *prdx-2* mutant strain displayed impaired locomotory activity (Figure S4I). The data demonstrates that physical fitness decreased with ageing and PRDX-2 is required for the enhanced locomotory activity or fitness in response to exercise.

In summary, the data presented demonstrate the adaptive rapid mitochondrial remodelling observed following acute exercise and ageing. PRDX-2 is sensitive to changes in the redox environment following exercise and required for exercise induced mitochondrial adaptations. Employing a cycle of acute exercise and recovery period at different ages in *C. elegans*, we observed that acute exercise initially induced mitochondrial fragmentation and mitophagy followed by mitochondrial fusion in young animals, but these responses decreased with age ultimately influencing physical fitness. Notably, the cycle of exercise and recovery failed to elicit these changes in the *prdx-2* mutant strain, evidenced by the failure to restore the redox environment, induce DAF-16 nuclear localisation and mitochondrial fusion, resulting in decreased physiological activity following exercise. Mechanistically, our results demonstrate that PRDX-2 is required for the nuclear localisation of DAF-16 following exercise induced ROS generation and subsequent changes in mitochondrial morphology. Collectively, our data identify the indispensable role of PRDX-2 in orchestrating mitochondrial dynamics in response to a physiological stress by regulating DAF-16.

## 4 Discussion

In this study, we employed the nematode *C. elegans* as a model to explore the mechanistic role of PRDX-2 in regulating mitochondrial dynamics during exercise and ageing. Our data demonstrate that exercise induced alterations in the redox environment, resulting in increased DAF-16 nuclear localisation and MERCS formation was dependent on PRDX-2, regulating mitochondrial remodelling and physiological activity. These signalling mechanisms are disrupted in ageing and with the loss of PRDX-2, where there was a failure to restore the redox environment and resulted in reduced mitochondrial fusion and increased mitochondrial fragmentation due to an inability to stimulate DAF-16 nuclear localisation.

Mitochondrial quality is intricately regulated by mitochondrial biogenesis and selective degradation, which collectively contribute to the maintenance of mitochondrial mass, morphology and size [4,31]. The dysregulation of mitochondrial biogenesis and impaired mitophagy are both recognised as contributing factors to compromised mitochondrial function in skeletal muscle during the ageing process [31,56,57]. Our previous studies have explored the effects of chronic swimming exercise on enhancing mitochondrial respiration, promoting mitochondrial biogenesis and regulating mitochondrial dynamics [23]. Recent research indicates that a cycle of mitochondrial fragmentation is induced by a single exercise session, followed by fusion after the recovery period [49]. This observation was corroborated by our current findings, which demonstrate that acute swimming triggers mitophagy, increases mitochondrial membrane potential and rapid changes in mitochondrial morphology. Furthermore, mitochondrial adaptations in response to exercise return to normal levels following a one-day recovery period. However, this cycle of mitochondrial remodelling was not observed in ageing worms as a result of an altered redox state of PRDX-2 and chronically elevated ROS, resulting in disrupted DAF-16 nuclear localisation and downstream signalling.

A single swim session induces locomotory fatigue, elevated mitochondrial ROS and increased mitochondrial metabolic rate [43]. Our data demonstrates that exercise-induced ROS was associated with specific changes in the redox state of PRDX-2, increased DAF-16 nuclear localisation and mitochondrial remodelling that increases physical fitness and resistance to oxidative stress in young worms. However, these alterations were delayed or not observed in old worms. Recent studies have proposed a theory termed “redox-stress response capacity (RRC)” to explain this phenomenon, suggesting that cellular signalling and homeostasis maintenance are regulated through ROS-mediated adaptive responses via a hormesis effect [58]. Furthermore, the decline in RRC over time was termed as redox-stress response resistance (RRR), which offers an explanation for the reduction in RRC observed during ageing [59]. Interestingly, the mechanisms of RRC and RRR were mediated by PRDX-2 and hyperoxidation of Peroxiredoxins [59]. Decreased H_2_O_2_ sensitivity of PRDX-2 and elevated PRDX-2 hyperoxidation have been observed in ageing human fibroblasts and *C. elegans* [59]. These findings align with our own observations, as evidenced by reversible changes in the redox state of PRDX-2 following exercise only in young worms and a corresponding increase in hyperoxidised Peroxiredoxins in old worms. Hyperoxidation of Peroxiredoxins can trigger alternate signalling pathways [60], for instance hyperoxidation of mammalian Prdx3 has been identified as translocating from mitochondria to the plasma membrane to inhibit cystine uptake during ferroptosis [61]. In *C. elegans* it has been suggested that hyperoxidised PRDX-2 has a chaperone function conferring stress resistance [50]. Furthermore, our previous redox proteomic analysis indicated that PRDX-2 was involved in a redox relay for the cellular adaptive response to exercise [23].

Peroxiredoxin 2, outcompetes other proteins for reaction with physiological concentrations of H_2_O_2_ due to its abundance and kinetic reactivity [62]. However, Peroxiredoxins have also been explored as involved in a redox relay for the transfer of oxidative equivalents to target proteins via reversible modification of cysteine residues on redox-sensitive proteins [23,59,63]. STAT3, NRF2, and FOXO (DAF-16) are key transcription factors involved in the regulation of mitochondrial function and muscle myogenesis [17,23,64]. Peroxiredoxin 2 has been identified to interact with STAT3, forming a redox relay in response to a short bolus of H_2_O_2_ treatment [65]. Peroxiredoxin 1 has also been implicated in binding and regulating the activity of FOXO3 [66]. Likewise, the nuclear localisation of DAF-16 can be induced by ROS, with evidence suggesting that this process is regulated by its redox-sensitive cysteine residues forming a mixed disulphide in a redox signalling cascade [16]. However, the precise relationship between PRDX-2 and DAF-16 remains ambiguous in the current understanding of cellular signalling pathways.

DAF-16 is the only ortholog of the FOXO transcription factors in *C. elegans* and vital for preserving cellular homeostasis, and it is required for the lifespan extension reported in *daf-2* mutant strains [15]. FOXO1 and FOXO3 govern glucose metabolism, lipid homeostasis and mitochondrial function primarily through their nuclear accumulation [67]. Elevated levels of ROS, originating from three mitochondrial mutants (*clk-1*, *isp-1* and *nuo-6*), prompt the nuclear translocation of DAF-16, thereby enhancing longevity [17]. The oxidation of cysteine residues within FOXO/DAF-16 are necessary for its nuclear localisation and subsequent activation in response to ROS [16]. A previous study demonstrated that DAF-16 exhibits increased nuclear localisation in response to elevated ROS levels, as well as following a swimming protocol [17]. Moreover, it is noteworthy that the heightened nuclear localisation of DAF-16 promotes mitochondrial fusion by increased EAT-3 expression through the inhibition of mitochondrial proteases *spg-7* and *ppgn-1* [18]. Our findings corroborate that acute exercise promotes DAF-16 nuclear accumulation, leading to an increase in mitochondrial dynamics. Furthermore, we observed increased expression levels of the mitophagy receptor *dct-1* and mitochondrial fusion regulator *eat-3* but decreased *ppgn-1* following acute exercise. Surprisingly, these changes were not observed in the *prdx-2* mutant strain, suggesting that PRDX-2 is required for maintaining mitochondrial quality via regulating DAF-16 in response to exercise.

The altered redox state of PRDX-2 following exercise can potentially trigger the downstream activation of SKN-1, which has been reported to occur via the p38 MAPK signalling cascade [68]. It is widely recognised that there are changes in the transcriptome, proteome and metabolome during the ageing process [8]. RNA sequencing data from isolated tissues of ageing *C. elegans* demonstrated elevated expression levels of *skn-1*, *daf-16* and *prdx-2* in body wall muscle [69]. Similarly, elevated levels of *daf-16* and *skn-1* were reported in adult D7 worms compared to adult D1 worms [70]. These findings are consistent with the results presented here, which demonstrate heightened SKN-1 reporter activity and increased PRDX-2 protein levels at D12 compared to D4 worms. The *prdx-2* mutant strain displays an accelerated ageing phenotype [23,50], characterised by a highly fragmented mitochondrial network evident at D4, which is aggravated with age. These findings suggest a potential role for PRDX-2 in maintaining mitochondrial integrity during both acute exercise and the ageing process. The depletion of PRDX-2 has previously been reported to enhance the activation of DAF-16 in response to low concentrations of arsenite [52]. These experiments were performed at the L4 stage in the *prdx-2* mutant strain. The accelerated ageing phenotype characteristic of the *prdx-2* mutant strain and altered redox environment could have resulted in the increased nuclear localisation of DAF-16 observed at this early developmental age.

In summary, our results demonstrate that there is blunted mitochondrial remodelling with age, which is associated with an altered redox environment. PRDX-2 was required for mitochondrial adaptations in response to exercise through the regulation of the intracellular redox environment and appropriate DAF-16 nuclear localisation. Our data demonstrate that during ageing, there are elevated levels of ROS, increased mitochondrial fragmentation, reduced survival capabilities and decreased locomotory activity. Employing a cycle of acute exercise and a recovery period model at various ages in *C. elegans*, acute exercise induces mitochondrial fragmentation and subsequent mitochondrial fusion through the activation of DAF-16, ultimately influencing physical fitness. Notably in the *prdx-2* mutant strain, the cycle of exercise and recovery failed to induce the observed changes compared to the N2 strain. The *prdx-2* mutant strain had an absence of alterations in mitochondrial membrane potential, mitochondrial dynamics as well as an inability to resolve the altered redox environment and reestablish mitochondrial adaptations and physiological activity following exercise. The redox state of PRDX-2 is sensitive to the redox environment and decreased reversible modification of the dimer ratio of PRDX-2 during ageing correlated with decreased survival and physical fitness in *C. elegans*. There are several limitations to the work presented in this study. We acknowledge that mitochondrial mass and morphology could affect the uptake of both MitoTracker Red and MItoSOX [71] used to estimate mitochondrial membrane potential and mitochondrial ROS generation, both of which are altered as a result of ageing and exercise. Furthermore, a physical interaction between PRDX-2 and DAF-16 was not demonstrated. DAF-16 forms a cysteine dependent interaction with IMB-2 (ortholog of Transportin-1) required for its nuclear translocation [16]. It would be interesting to determine if PRDX-2 facilitates this interaction directly in a redox relay mechanism or as a result of the regulation of the redox environment for appropriate DAF-16 activation. As demonstrated, exercise induced ROS generation can activate a number of redox sensitive signalling pathways including DAF-16 and SKN-1, that can affect mitochondrial function through a number of mechanisms with subsequent effects on stress resilience and longevity. Similarly, the acute exercise intervention performed in this study was initially with D4 adults, the *prdx-2* mutant already possesses a disrupted mitochondrial morphology at this age. It would be informative to perform these experiments using a strain with a normal mitochondrial morphology using a diluted RNAi approach to reduce PRDX-2 expression. Despite these limitations, this study demonstrates the pivotal role of PRDX-2 in regulating appropriate DAF-16 nuclear localisation and mitochondrial remodelling following acute ROS generation during exercise and ageing.

## CRediT authorship contribution statement

**Qin Xia:** Writing – review & editing, Writing – original draft, Visualization, Methodology, Formal analysis, Conceptualization. **Penglin Li:** Writing – review & editing, Methodology, Formal analysis. **José C. Casas-Martinez:** Writing – review & editing, Methodology, Formal analysis. **Antonio Miranda-Vizuete:** Writing – review & editing, Resources, Investigation. **Emma McDermott:** Writing – review & editing, Resources, Methodology. **Peter Dockery:** Writing – review & editing, Resources, Methodology, Formal analysis. **Katarzyna Goljanek-Whysall:** Writing – review & editing, Supervision, Resources, Methodology. **Brian McDonagh:** Writing – review & editing, Writing – original draft, Supervision, Resources, Methodology, Formal analysis, Conceptualization.

## Declaration of competing interest

The authors declare that they have no known competing financial interests or personal relationships that could have appeared to influence the work reported in this paper.

## Data Availability

Source data is available on Mendeley Data DOI: 10.17632/shysb8dsw3.1.
