# Supplementary material for: Peroxiredoxin 2 regulates DAF-16/FOXO mediated mitochondrial remodelling in response to exercise that is disrupted in ageing
Source: Mol Metab. 2024 Aug 6;88:102003. doi: 10.1016/j.molmet.2024.102003 (PMC11388264; doi:10.1016/j.molmet.2024.102003)
Supplement: Multimedia component 1 [file mmc1.docx]

**Supplementary Figures and Tables**


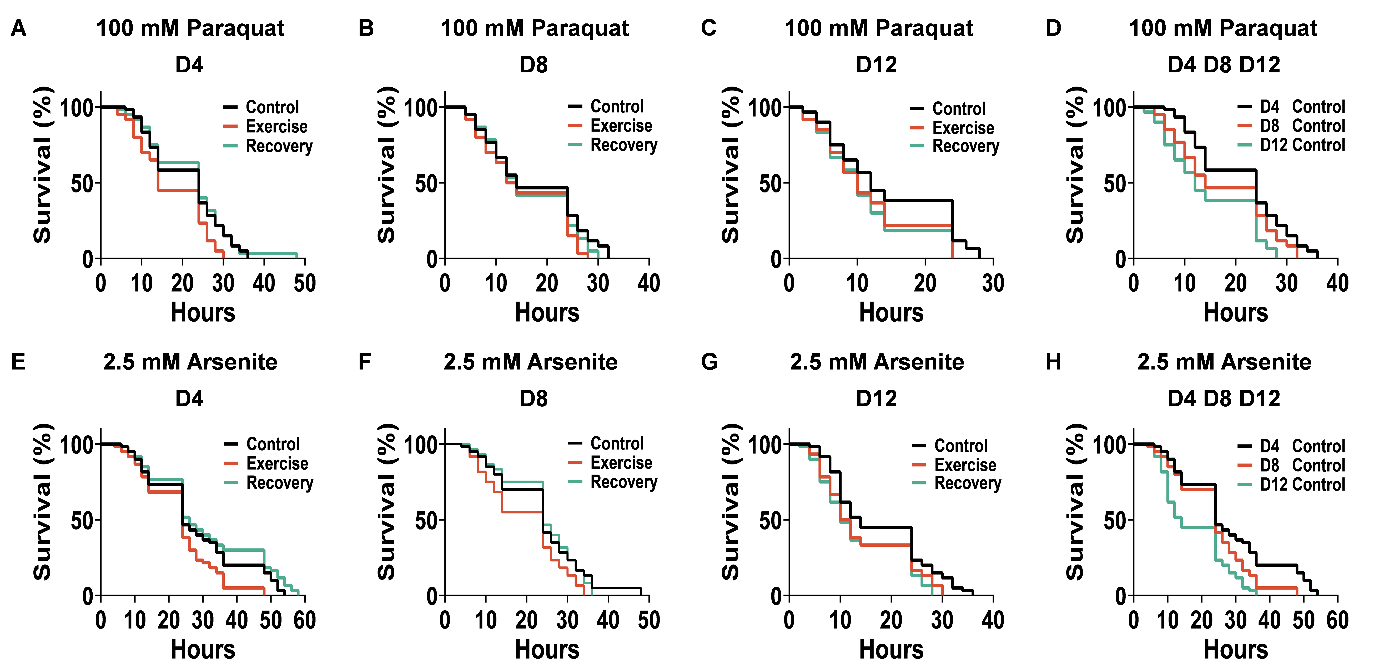


**Figure S1** **Decay of resistance to paraquat and arsenite with ageing and exercise.**

(A-D) Decreased survival to paraquat following acute exercise and ageing (A-D), n= 60.

(E-H) Decreased survival to arsenite following acute exercise and ageing (E-H), n = 60.

The Log-rank (Mantel-Cox) test was employed to compare survival between distinct groups. *p* values (A: C vs E *p*= 0.0034, E vs R *p*= 0.001; B: C vs E *p*= 0.0435; C: C vs E *p*= 0.022, C vs R *p*= 0.0076; D: D4 vs D8: *p*= 0.0266; D4 vs D12: *p* < 0.0001; D8 vs D12: *p*= 0.0181. E: C vs E *p*= 0.0114, E vs R *p*= 0.0009; F: C vs E *p*= 0.0143, E vs R *p*= 0.0079; G: C vs E *p*= 0.031; C vs R *p*= 0.0048; H: D4 vs D8: *p*= 0.0136; D4 vs D12: *p* < 0.0001; D8 vs D12: *p*= 0.0023).


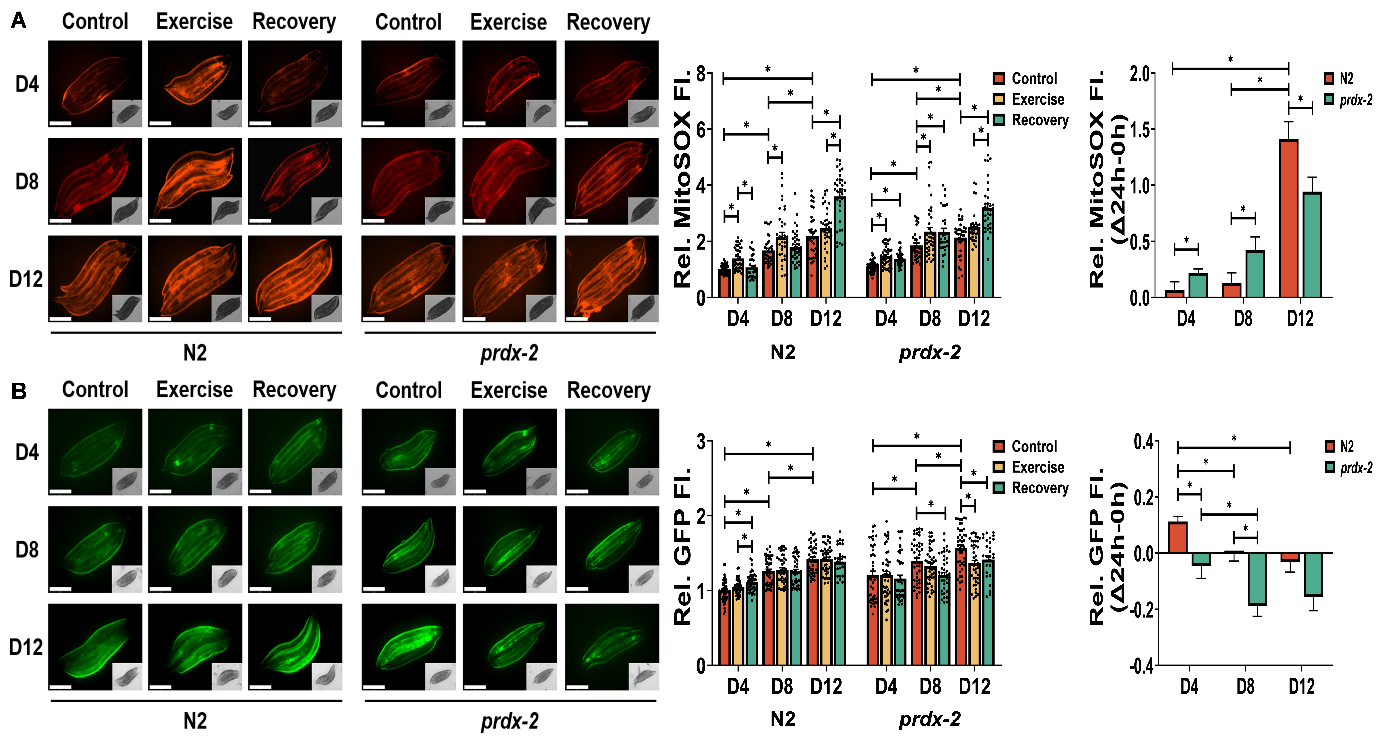
**Figure S2 Ageing and the loss of PRDX-2 directly affect MitoSOX staining and SKN-1 activation.** (A-B) MitoSOX red staining of worms for mitochondrial ROS (A) and *gst-4p::gfp* SKN-1 transcriptional reporter (B) following acute exercise at different stages, scale bar = 275 μm, n= 30-40.

Graphs are the normalised relative means ± SEM and *p*-value of < 0.05 was considered as statistically significant *(*p* < 0.05), one-way or two-way ANOVA was used for significance between groups (a–b). *p* values (a: in N2 worms: D4: C vs E < 0.0001, E vs R = 0.0004; D8: C vs E = 0.0094; D12: C vs R < 0.0001, T vs R < 0.0001; D4 vs D8 < 0.0001; D4 vs D12 < 0.0001; D8 vs D12 = 0.0004; in *prdx-2* worms: D4: C vs E < 0.0001, C vs R = 0.0004; D8: C vs E = 0.0163, C vs R = 0.0315; D12: C vs T < 0.0001, E vs R = 0.0001; D4 vs D8 < 0.0001; D4 vs D12 < 0.0001; D8 vs D12 = 0.0332; recovery rate: N2: D4 vs D12 < 0.0001; D8 vs D12 < 0.0001; *prdx-2*: D4 vs D12 < 0.0001; D8 vs D12 = 0.0013; N2 vs *prdx-2*: D4 = 0.0453; D8 = 0.0486; D12 = 0.0211. b: in N2 worms: D4: C vs R < 0.0001, E vs R = 0.0217; D4 vs D8 < 0.0001; D4 vs D12 < 0.0001; D8 vs D12 < 0.0001. in *prdx-2* worms: D8: C vs R = 0.0024; D12: C vs E = 0.0014, C vs R = 0.045; D4 vs D8 = 0.0137; D4 vs D12 < 0.0001; D8 vs D12 = 0.0197. recovery rate: N2: D4 vs D8 = 0.0035; D4 vs D12 = 0.0007; *prdx-2*: D4 vs D8 = 0.0448; N2 VS *prdx-2*: D4 = 0.0018; D8 = 0.0001)


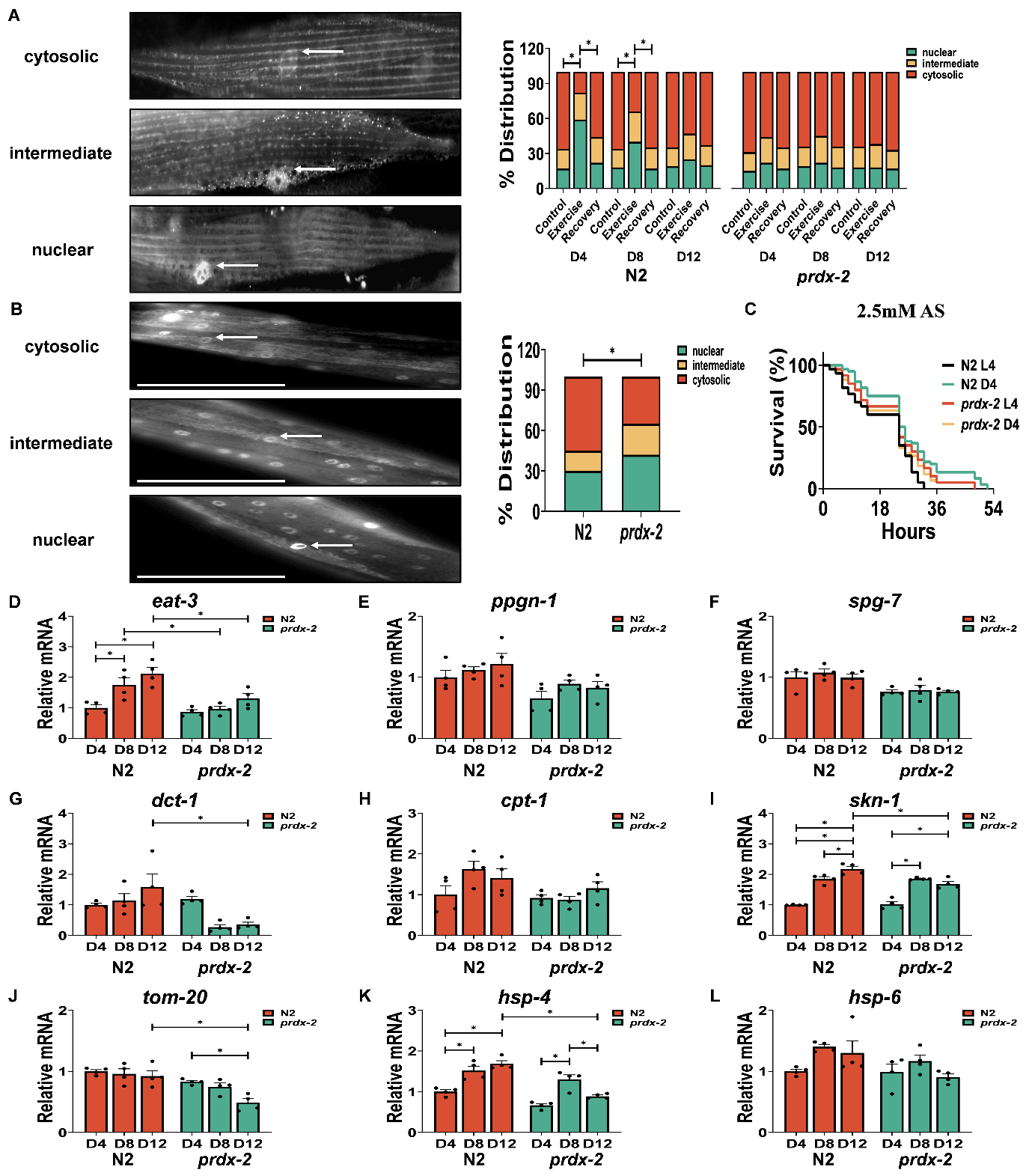
 **Figure S3 Ageing and loss of PRDX-2 in adult *C. elegans* adversely impacts DAF-16 nuclear localisation and mitochondrial dynamics in response to exercise.** (A-B) Representative images of the TJ356 strain DAF-16::GFP distribution at adult (A) and L4 (B) stage in *C. elegans,* scale bar = 50 μm, n = 130-150. (C) Survival to arsenite in *prdx-2* mutant compared to N2 at L4 and D4 stage, n = 60. (D-L) mRNA level of *eat-3* (D), *ppgn-1* (E), *spg-7* (F), *dct-1* (G), *cpt-1* (H), *skn-1* (I), *tom-20* (J), *hsp-4* (K) and *hsp-6* (L) at different stages, n = 4. Graphs are the normalised relative means ± SEM and *p*-value of < 0.05 was considered as statistically significant *(*p* < 0.05). *p* values (A: in N2 worms: D4: C vs E < 0.0001, E vs R < 0.0001; D8: C vs E < 0.0001, E vs R < 0.0001. B: N2 vs *prdx-2* = 0.0172. C: N2 L4 vs *prdx-2* L4 = 0.0094; N2 D4 vs *prdx-2* D4 = 0.0186. D: in N2 worms: D4 vs D8 = 0.0274; D4 vs D12 = 0.0007. N2 vs *prdx-2*: D8 = 0.0188, D12 = 0.0135. G: N2 vs prdx-2: D12 = 0.0052. I: in N2 worms: D4 vs D8 < 0.0001, D4 vs D12 < 0.0001, D8 vs D12 = 0.0355; in *prdx-2*: D4 vs D8 < 0.0001, D4 vs D12 < 0.0001. N2 vs *prdx-2*: D12 = 0.0011. J: in *prdx-2* worms: D4 vs D12 = 0.0126; N2 vs *prdx-2*: D12 = 0.0013. K: in N2 worms: D4 vs D8 = 0.002, D4 vs D12 < 0.0001; in *prdx-2* worms: D4 vs D8 = 0.0002, D8 vs D12 = 0.0154; N2 vs *prdx-2*: D12: < 0.0001)


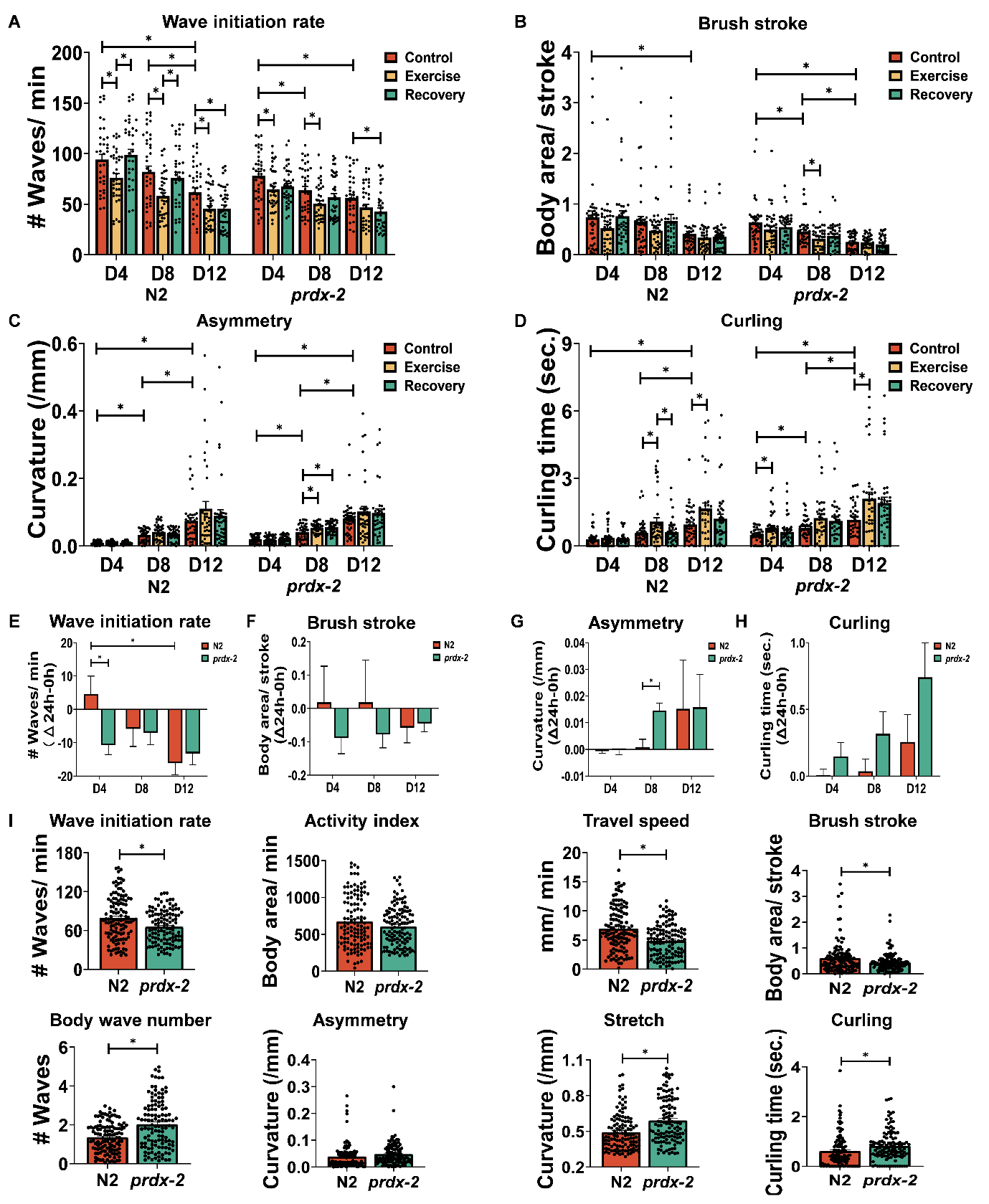
**Figure S4 Ageing and loss of PRDX-2 undermines physical fitness following exercise.**

(A-D) Wave initiation rate (A), brush stroke (B), asymmetry (C) and curling (D) following acute exercise at different stages, n = 30-40.

(E-H) The recovery rate of wave initiation rate (E), brush stroke (F), asymmetry (G) and curling (H) compared to normal condition, n = 30-40.

(I) Physical fitness overall 12 days of N2 and *prdx-2* worms, n = 90-120.

Graphs are the normalised relative means ± SEM and *p*-value of < 0.05 was considered as statistically significant *(*p* < 0.05). *p* values (A: in N2 worms: D4: C vs E = 0.027, E vs R = 0.0037; D8: C vs E = 0.0019, E vs R = 0.0278; D12: C vs E = 0.0063, C vs R = 0.0056; D4 vs D12 < 0.0001; D8 vs D12 = 0.014; in *prdx-2* worms: D4: C vs E = 0.0141; D8: C vs E = 0.022; D12: C vs R = 0.0179; D4 vs D8 = 0.0196; D4 vs D12 = 0.0001. B: in N2 worms: D4 vs D12 = 0.0433. in *prdx-2* worms: D8: C vs E = 0.0432; D4 vs D8 = 0.0318; D4 vs D12 < 0.0001; D8 vs D12 = 0.0133. C: in N2 worms: D4 vs D8 = 0.0164; D4 vs D12 < 0.0001; D8 vs D12 < 0.0001; in *prdx-2* worms: D8: C vs E = 0.016, C vs R = 0.0036; D4 vs D8 = 0.017; D4 vs D12 < 0.0001; D8 vs D12 < 0.0001. D: in N2 worms: D8: C vs E = 0.0161, E vs R = 0.0303; D12: C vs E = 0.0277; D4 vs D12 < 0.0001; D8 vs D12 = 0.013; in *prdx-2* worms: D4: C vs E = 0.0369; D12: C vs E = 0.014; D4 vs D8 = 0.0327; D4 vs D12 < 0.0001; D8 vs D12 = 0.0087. E: in N2 worms: D4 vs D12 = 0.0066; N2 vs *prdx-2*: D4 = 0.0126. G: N2 vs *prdx-2*: D8 = 0.001. I: wave initiation rate: N2 vs *prdx-2* = 0.0007; travel speed: N2 vs *prdx-2* < 0.0001; brush stroke: N2 vs *prdx-2* = 0.015; body wave number: N2 vs *prdx-2* < 0.0001; stretch: N2 vs *prdx-2* < 0.0001; curling: N2 vs *prdx-2* = 0.0098)

**Table S1** Data from oxidative stress assays related to Figure 1B, 1C and S3C.

| Figures | age | Condition | n | Mean lifespan | Log-rank test, *p*-value |
| --- | --- | --- | --- | --- | --- |
| Figure 1B | D4 | Control | 60 | 21.1 ± 1.14 |  |
|  |  | Exercise | 60 | 17.33 ± 1.05 | compared to D4 C, *p*= 0.0034 |
|  |  | Recovery | 60 | 21.83 ± 1.19 | compared to D4 E, *p*= 0.0011 |
|  | D8 | Control | 60 | 17.57 ± 1.19 | compared to D4 C, *p*= 0.0266 |
|  |  | Exercise | 60 | 15.8 ± 1.08 | compared to D8 C, *p*= 0.0435 |
|  |  | Recovery | 60 | 16.5 ± 1.09 | compared to D8 E, *p*= 0.2379 |
|  | D12 | Control | 60 | 14.7 ± 1.11 | compared to D8 C, *p*= 0.0181 |
|  |  | Exercise | 60 | 11.83 ± 0.93 | compared to D12 C, *p*= 0.022 |
|  |  | Recovery | 60 | 11.27 ± 0.89 | compared to D12 C, *p*= 0.0076 |
| Figure 1C | D4 | Control | 60 | 28.2 ± 1.97 |  |
|  |  | Exercise | 60 | 23.37 ± 1.34 | compared to D4 C, *p*= 0.0114 |
|  |  | Recovery | 60 | 30.83 ± 2 | compared to D4 E, *p*= 0.0009 |
|  | D8 | Control | 60 | 23.77 ± 1.34 | compared to D4 C, *p*= 0.0136 |
|  |  | Exercise | 60 | 19.67 ± 1.19 | compared to D8 C, *p*= 0.0143 |
|  |  | Recovery | 60 | 24.03 ± 1.12 | compared to D8 E, *p*= 0.0079 |
|  | D12 | Control | 60 | 17.77 ± 1.21 | compared to D8 C, *p*= 0.0023 |
|  |  | Exercise | 60 | 14.6 ± 1.12 | compared to D12 C, *p*= 0.0231 |
|  |  | Recovery | 60 | 13.93 ± 1.08 | compared to D12 C, *p*= 0.0048 |
| Figure S3C | L4 | N2 | 60 | 19.3 ± 1.23 |  |
|  |  | *prdx-2* | 60 | 22.9 ± 1.43 | compared to N2 L4, *p*= 0.0094 |
|  | D4 | N2 | 60 | 26.47 ± 1.57 |  |
|  |  | *prdx-2* | 60 | 21.77 ± 1.43 | compared to N2 D4, *p*= 0.0186 |

Mean lifespan and statistical analysis were determined by OASIS2 platform, Kaplan-Meier curve was performed using GraphPad Prism 7.

**Table S2** genotyping and qPCR primers

| Oligonucleotides | Source | primer |
| --- | --- | --- |
| Genotyping | | |
| prdx-2 | Sigma | OF: CGCTCCTCCTAAACGTTGTAGTC  OR: CTAGGGATACGGGGGAAATTAG  IR: GGATATTGAAACCCAACGGCAACG |
| qPCR | | |
| *prdx-2* | Sigma | Forward: CATTCCAGTTCTCGCTGAC  Reverse: ATGATGAAGAGTCCACGGA |
| *prdx-3* | Sigma | Forward: GTTCCGTTCTCTTGGAGCTG  Reverse: CTTGTTGAAATCAGCGAGCA |
| *prdx-6* | Sigma | Forward: GGAGAACAATGCTGATGC  Reverse: ATCTGAACATGGCGTTTGC |
| *skn-1* | Sigma | Forward: GGACAACAGAATCCCAAAGG  Reverse: TCAGGACGTCAACAGCAGAC |
| CDC-42 | Sigma | Forward: AGCCATTCTGGCCGCTCTCG  Reverse: GCAACCGCTTCTCGTTTGGC |
| *eat-3* | Sigma | Forward: AGAGCATCGAAACCGGATGG  Reverse: GCGTCAGCATAGCTTCTTCG |
| *ppgn-1* | Sigma | Forward: ATGCTTCTACACCGCTCCAC  Reverse: GTGGAAATCTGCGAGCACT |
| *spg-7* | Sigma | Forward: CCGTTGTCGTTTGAGACACC  Reverse: CGGCGAAGTGCGTTCATTAC |
| *cpt-1* | Sigma | Forward: AACGACGTACCGGACGATTC  Reverse: CCGAGGTAGCCATCACCAAA |
| *dct-1* | Sigma | Forward: GAAAATACACCTCCAAAGACTGTCCG  Reverse: GATGTTTGATTAGTTTCCGGCAAACAG |
| *hsp-4* | Sigma | Forward: ACGACCACAATCGTCTCAGTCC  Reverse: CTTCGTCAGTGAGCTTTCCTCC |
| *hsp-6* | Sigma | Forward: GAACCGGAAAGGAACAACAGATCG  Reverse: GCAATCTTGGTTCGGAGAGCCTC |
| *tomm-20* | Sigma | Forward: CGGCTACTGCATTTACTTCGA  Reverse: TCATTGCCTGCTGCAGCTGGA |

**Reagents and Resources:**

| REAGENT or RESOURCE | SOURCE | IDENTIFIER |
| --- | --- | --- |
| **Antibodies** | | |
| rabbit anti-Peroxiredoxin 2 (*C. elegans*) | Elizabeth Veal lab [65] | N/A |
| rabbit anti-Peroxiredoxin-SO_3_ | Abcam | Cat# ab16830, RRID:AB_443491 |
| IRDye 800CW Goat anti-Rabbit IgG | LI-COR Biosciences | Cat# 926-32211, RRID:AB_621843 |
| **Bacterial and Virus Strains** | | |
| *E. coli*: Strain OP50 | Caenorhabditis Genetics Center | N/A |
| **Chemicals, Peptides and Recombinant Proteins** | | |
| Acetic acid | Sigma | Cat# A6283 |
| Acrylamide | Sigma | Cat# A3699 |
| Agar | Sigma | Cat# A1296 |
| APS | Sigma | Cat# A3678 |
| Arsenite | Sigma | Cat# S7400 |
| Beta-mercaptoethanol | Sigma | Cat# M7522 |
| Boric acid | Sigma | Cat# B0252 |
| Bradford Reagent | Bio-Rad | Cat# 5000006 |
| Bromophenol blue | Sigma | Cat# 114391 |
| BSA | Sigma | Cat# A3059 |
| CaCl_2_ | Sigma | Cat# C1016 |
| Chloroform | Sigma | Cat# C0549 |
| Cholesterol | Sigma | Cat# C8667 |
| dATP | Thermo Fisher Scientific | Cat# R0141 |
| dCTP | Thermo Fisher Scientific | Cat# R0151 |
| dGTP | Thermo Fisher Scientific | Cat# R0161 |
| DTT | Sigma | Cat# Y00147 |
| dTTP | Thermo Fisher Scientific | Cat# R0171 |
| EDTA | Sigma | Cat# ED2SS |
| Ethanol | Sigma | Cat# E7023 |
| Glycerol | Sigma | Cat# G6279 |
| Glycine | Sigma | Cat# G8898 |
| Isopropanol | Sigma | Cat# I9516 |
| K_2_HPO_4_ | Sigma | Cat# 3786 |
| KH_2_PO_4_ | Sigma | Cat# P9791 |
| Levamisole | Sigma | Cat# L9756 |
| Methanol | Sigma | Cat# 34860 |
| MgSO_4_ | Sigma | Cat# M7506 |
| MyTaq ™ Reaction Buffer Red | Sigma | Cat# R2523 |
| Na_2_HPO_4_ | Sigma | Cat# S0876 |
| NaCl | Sigma | Cat# S9888 |
| NaClO | Sigma | Cat# 1056142500 |
| NaOH | Sigma | Cat# S5881 |
| NEM | Sigma | Cat# E3876 |
| Nitrocellulose Blotting membrane | GE Healthcare Life Sciences | Cat# 1060003 |
| Nystatin | Sigma | Cat# N3503 |
| Paraquat | Sigma | Cat# 856177 |
| Peptone | Sigma | Cat# 91249 |
| Ponceau S Staining Solution | Thermo Fisher Scientific | Cat# A40000279 |
| Protease Inhibitor Cocktail | Sigma | Cat# P8340 |
| Proteinase K | Sigma | Cat# 03115828001 |
| Random hexamer | Sigma | Cat# 100026484 |
| Ribolock | Thermo Fisher Scientific | Cat# EO0381 |
| RNase free water | Sigma | Cat# W4502 |
| RT buffer | Sigma | Cat# Y02321 |
| SDS | Sigma | Cat# L3771 |
| Superscript II | Sigma | Cat# 100004925 |
| SYBR Green | Qiagen | Cat# 339347 |
| TEMED | Sigma | Cat# T9281 |
| Triton^TM^ X-100 | Sigma | Cat# T8787 |
| Trizma® base | Sigma | Cat# T1503 |
| TRIzol Reagent | Life Technologies | Cat# 15596018 |
| UltraPure ^TM^ Agarose | Sigma | Cat# 16500 |
| **Critical Commercial Assays** | | |
| MitoTracker^TM^ Red CMXRos | Thermo Fisher Scientific | Cat# M7512 |
| MitoSOX^TM^ Red mitochondrial superoxide indicator | Thermo Fisher Scientific | Cat# M36008 |
| Carboxy-H_2_DCFDA | Thermo Fisher Scientific | Cat# C2938 |
| **Experimental Models: Organisms/Strains** | | |
| *C. elegans*: wild type | Caenorhabditis Genetics Center | N2, RRID:WB-STRAIN:WBStrain00000001 |
| *C. elegans: prdx-2* VE1 | Elizabeth A Veal | VE1, Backcrossed with *gk169* RRID:WB-STRAIN:WBStrain00035650 |
| *C. elegans: dvIs19[(pAF15)gst-4p::gfp] III* | Caenorhabditis Genetics Center | CL2166, RRID:WB-STRAIN:WBStrain00005102 |
| *C. elegans: zcIs14 [myo-3::gfp(mit)]* | Caenorhabditis Genetics Center | SJ4103, RRID:WB-STRAIN:WBStrain00034069 |
| *C. elegans*: *I (unc-119(ed3);*  *Ex[myo-3p tomm20::Rosella;unc-119(+)]* | Tavernarakis Lab | IR2539 |
| C. elegans: *zIs356 [daf-16p::daf-16a/b::GFP + rol-6(su1006)]* | Miranda-Vizuete Lab | TJ356, RRID:WB-STRAIN:WBStrain00034892 |
| C. elegans: *daf-16(ot971[daf-16::GFP]) I.* | Miranda-Vizuete Lab | OH16024 |
| *C. elegans: prdx-2* (VE1)*; dvIs19[(pAF15)gst-4p::GFP::NLS] III* | This paper | *prdx-2* x CL2166 |
| *C. elegans: prdx-2* (VE1)*; zcIs14 [myo-3::GFP(mit)]* | This paper | *prdx-2* x SJ4103 |
| *C. elegans: prdx-2* (VE1)*; unc-119(ed3); Ex[pmyo-3TOMM-20::Rosella;unc-119(+)]* | This paper | *prdx-2* x IR2539 |
| *C. elegans: prdx-2* (VE1)*; zIs356 [daf-16p::daf-16a/b::GFP + rol-6(su1006)]* | This paper | *prdx-2* x TJ356 |
| *C. elegans: prdx-2* (VE1) *; daf-16(ot971[daf-16::GFP]) I.* | This paper | *prdx-2* x OH16024 |
| **Oligonucleotides** | | |
| See Table S2 | | |
| **Software and Algorithms** |  |  |
| Prism 7 | GraphPad Software | RRID:SCR_002798 |
| ImageJ | NIH | RRID:SCR_003070 |
| Image Studio Lite | Image Studio Lite | RRID:SCR_013715 |
| CeLeSt | Restif et al., 2014 [57] | N/A |
| OASIS 2 | Online Application for Survival Analysis 2 | N/A |
